# Neuronal development in the cochlea of a nonhuman primate model, the common marmoset

**DOI:** 10.1002/dneu.22850

**Published:** 2021-10-22

**Authors:** Makoto Hosoya, Masato Fujioka, Ayako Y Murayama, Hiroyuki Ozawa, Hideyuki Okano, Kaoru Ogawa

**Affiliations:** ^1^ Department of Otorhinolaryngology Head and Neck Surgery Keio University School of Medicine Tokyo Japan; ^2^ Department of Physiology Keio University School of Medicine Tokyo Japan; ^3^ Laboratory for Marmoset Neural Architecture Center for Brain Science RIKEN Wako Japan

**Keywords:** cochlea, common marmoset, inner ear, primate

## Abstract

Precise cochlear neuronal development is vital to hearing ability. Understanding the developmental process of the spiral ganglion is useful for studying hearing loss aimed at aging or regenerative therapy. Although interspecies differences have been reported between rodents and humans, to date, most of our knowledge about the development of cochlear neuronal development has been obtained from rodent models because of the difficulty in using human fetal samples in this field. In this study, we investigated cochlear neuronal development in a small New World monkey species, the common marmoset (*Callithrix jacchus*). We examined more than 25 genes involved in the neuronal development of the cochlea and described the critical developmental steps of these neurons. We also revealed similarities and differences between previously reported rodent models and this primate animal model. Our results clarified that this animal model of cochlear neuronal development is more similar to humans than rodents and is suitable as an alternative for the analysis of human cochlear development. The time course established in this report will be a useful tool for studying primate‐specific neuronal biology of the inner ear, which could eventually lead to new treatment strategies for human hearing loss.

## 1 INTRODUCTION

Auditory perception is the process by which mechanical sound waves are detected by the inner ear and converted into neuronal electrical impulses that are perceived by the brain. The inner ear is a peripheral sensory organ for hearing and equilibrium. In mammals, the inner ear can be divided into two parts: the cochlea for the sense of hearing and the vestibule/semicircular canal for equilibrium. In the cochlea, hair cells convert mechanosensory sound waves into neural electrical pulses. These pulses eventually reach the auditory cortex of the brain, where we can perceive sound stimuli via auditory neurons. This afferent transmission of neural electrical pulses is achieved by sophisticated interaction between the inner hair cells and spiral ganglion neurons. This process is tuned simultaneously by the efferent neuronal pulse from the brain to the cochlea or by electrically driven motility of the outer hair cells.

Fine‐tuned neuronal development of spiral ganglion neurons in the cochlea is essential for healthy hearing acquisition. This development requires several well‐controlled steps, including innervation of neurites to the sensory epithelium by neurotrophic signals, synaptic formation between the hair cells and spiral ganglion neurons, maturation of synaptic activity and myelination, and pruning of the excess neurite.

Most of our knowledge about cochlear neuronal development has been obtained from rodent models, specifically mice and rats. However, to date, several interspecies differences in neuronal development between rodents and humans have been reported. For example, the expression pattern of peripherin (PRPH), which is a well‐known type II spiral ganglion neuron marker, was different between humans and rodents in the cochlear development process (Locher et al., 2013). The interspecies difference was also reported for myelination of spiral ganglion: in humans, most spiral ganglion neurons lack a myelin layer around their perikaryon, whereas in mice the perikaryon is surrounded by a myelin sheath, and this lack of myelination may enable the close physical interaction between neural elements in humans (Glueckert et al., 2005; Ota & Kimura, 1980; M. R. Romand & R. Romand, 1987). This means that our knowledge about cochlear neuronal development obtained from rodent models cannot always be applied directly to humans. Despite the reported interspecies differences between rodents and humans, there is little knowledge regarding the neuronal development of humans. This is because most of the cochlear neuronal development occurs in the relatively late phase of human gestation (around GW20) and obtaining and using well‐prepared fetus samples of such stages is extremely difficult due to ethical problems.

To overcome this limitation in sampling and to reduce or minimize species differences, the small primate model animal, the common marmoset (*Callithrix jacchus*), has been used in vocal communication (Eliades & Miller, 2017; Gultekin & Hage, 2017; Osmanski et al., 2013; X. Wang & Kadia, 2001), audiological (L. A. Johnson et al., 2012, 2016), and neuroscience research (Okano, 2021). Recently, this species has also been used in genetic hearing loss research (Hosoya et al., 2016; Hosoya et al., 2016; Matsuzaki et al., 2018; N. Suzuki et al., 2016). Moreover, genetic modification is now possible in the common marmoset (Sasaki et al., 2009).

We previously reported general information about cochlear development in common marmosets, including basic anatomical staging compared to humans and mice, as well as expression patterns of conventional markers of hair cells, supporting cells, spiral ganglion neurons, and other cochlear cells (Hosoya et al., 2021). Moreover, we reported that there are several differences in the development of hair cells and spiral ganglion neurons between rodents and the marmoset, which is more similar to human development.

Additionally, understanding the developmental process of the nonhuman primate cochlea is important for both clinical and basic research. Knowledge about organ development is important from a clinical viewpoint, such as investigating organ regeneration, because the developmental steps and regenerative process possess common mechanisms in many organs. Therefore, understanding human cochlear neuronal development is necessary for the advancement of regenerative medicine of the inner ear. Recently, hearing disability due to inner hair cell‐spiral ganglion neuron synaptopathy is of great interest in this field. It has been described as "hidden hearing loss," as it is not thought to be detectable using standard measures of the audiometric threshold (Kujawa & Liberman, 2009). The prevalence of this type of dysfunction has been estimated to be 12–15% (Spankovich et al., 2018; Tremblay et al., 2015). To overcome this type of hearing loss either regeneration or reformation of the synapse may be a feasible treatment. However, our knowledge about human cochlear neurons is limited, although there are several known interspecies differences between humans and rodents. Establishing a novel platform for investigating cochlear neuronal development, which can bridge the gap between human and rodent models, is critical for developing a treatment for cochlear synaptopathy.

For these reasons, it is necessary to examine in detail the neuronal development of the common marmoset to understand the primate‐specific neuronal development of the inner ear. Here, we report the detailed description of cochlear development of the nonhuman primate model.

## 2 RESULTS AND DISCUSSION

### 2.1 Expression pattern of GAP43 and MAP2 in the marmoset cochlear development

Neuronal development of spiral ganglion neurons and their connection to the hair cells starts with neurite outgrowth toward the sensory epithelium. First, we examined early neurite outgrowth in a common marmoset with immature neurite markers growth‐associated protein 43 (GAP43) and microtubule‐associated protein 2 (MAP2).

GAP43 is a major protein kinase C substrate of growth cones and developing nerve terminals (Skene, 1989). GAP43 is associated with presynaptic neuronal outgrowth and neuronal plasticity by assisting neuronal pathfinding and branching during development and regeneration (Skene, 1989). GAP43 is highly expressed during the early stages of development in innervating growth cones of neurites, and it is used as an indicator of neurite elongation and synapse formation. Upon maturation of the neurites, GAP43 is downregulated by most neurons. GAP43 can be used as a marker for neuronal regeneration. Thus, this preservation of expression patterns between rodents and primates may indicate the usefulness of GAP43 as a marker of cochlear neuronal regeneration.

**FIGURE 1:**
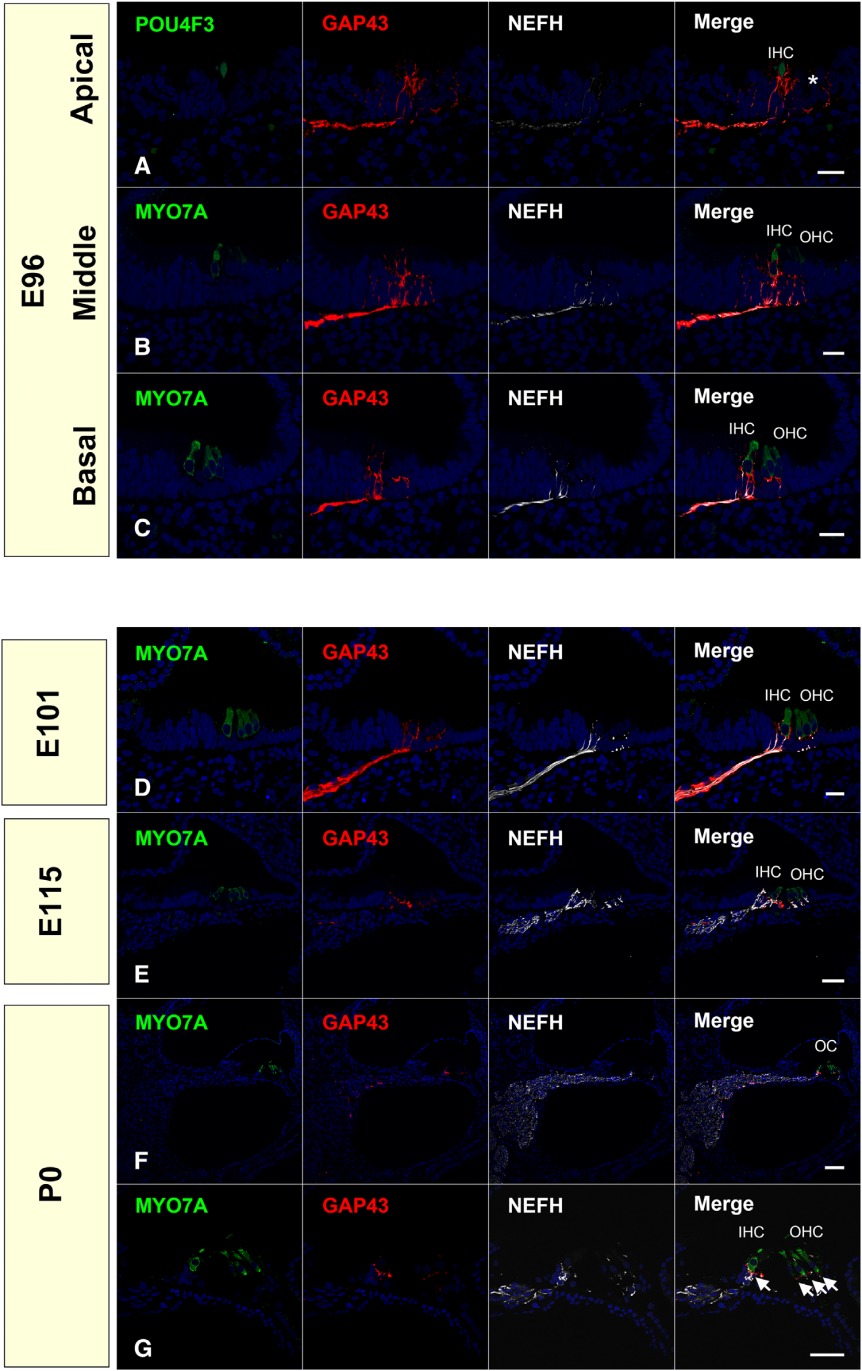
Changes of expression patterns of GAP43 during development. (a)–(c) GAP43 was expressed in the prosensory domain at E96. In the apical turn, GAP43 expression can be observed in the region under the immature hair cells, followed by MYO7a expression in the outer hair cells (asterisk in a). (d) At E101, GAP43 expression was broadly observed in the spiral ganglion neuron. (e)–(g) After E115, GAP43 expression comes to be restricted more peripherally in the axon terminal of the spiral ganglion neurons. (f) and (g) At P0, only a slight expression was observed in the neurite's tip under the organ of Corti (arrow in g). Nuclei were counterstained with Hoechst stain (blue). Scale bar: 20 μm in (a)–(d), 50 μm in (e) and (g), 100 μm in (f). IHC, inner hair cells; OHC, outer hair cells. OC: organ of Corti

In this study, we observed GAP43 expression in the prosensory domain of the E96 marmoset embryo (Figure 1a–c). At this stage, strong GAP43 expression was observed in all turns of the cochlea. Especially in the apical turn, we observed a low expression of POU4F3 (POU class 4 homeobox 3) in the positive inner ear and none in the outer hair cell region; however, GAP43 expression was broadly observed in the POU4F3 negative outer hair cell region (Figure 1a). This indicates that the GAP43 positive neurite growth cone of spiral ganglion neurons was innervated before developing mature hair cells and reached near the future hair cells before POU4F3 or MYO7A (Myosin7A) expression in the hair cells. After E101, GAP43 expression gradually decreased (Figure 1d–g), and finally at P0 (Postnatal Day = 0), only a slight GAP43 expression was observed at the tip of the axon terminal of the spiral ganglion neurons in the organ of Corti (Figures 1f,g).

To date, several reports have described the expression patterns of GAP43 during cochlear development (Bartolome et al., 2002; Knipper et al., 1995; Merchan‐Perez et al., 1993). Previously, Bartolome et al. (2002) reported that GAP43 immunoreactivity stains afferent neurites and cochlear ganglion cell bodies of the rat cochlea. They reported that in rats, from E13 to E18, immunoreactivities can be detected in the ganglion neuron perikarya of the afferent neuron, and GAP43 can be detected in nerve fibers inside the undifferentiated sensory epithelium at E18. GAP43 immunoreactivity is restricted within the intraganglionic spiral bundle (IGSB) and the efferent nerve fibers reaching the cochlear epithelium ganglion (E18‐P12 in rats). In the rodent cochlea, temporal GAP43 expression has also been reported in developing efferent nerves (Knipper et al., 1995). These expression patterns are well preserved between rodents and common marmosets. It is known that GAP43 can be used as a marker of neuronal regeneration. Thus, this preservation of expression pattern between the rodents and a primate may indicate the usefulness of GAP43 as a marker of cochlear neurogenesis.

**FIGURE 2:**
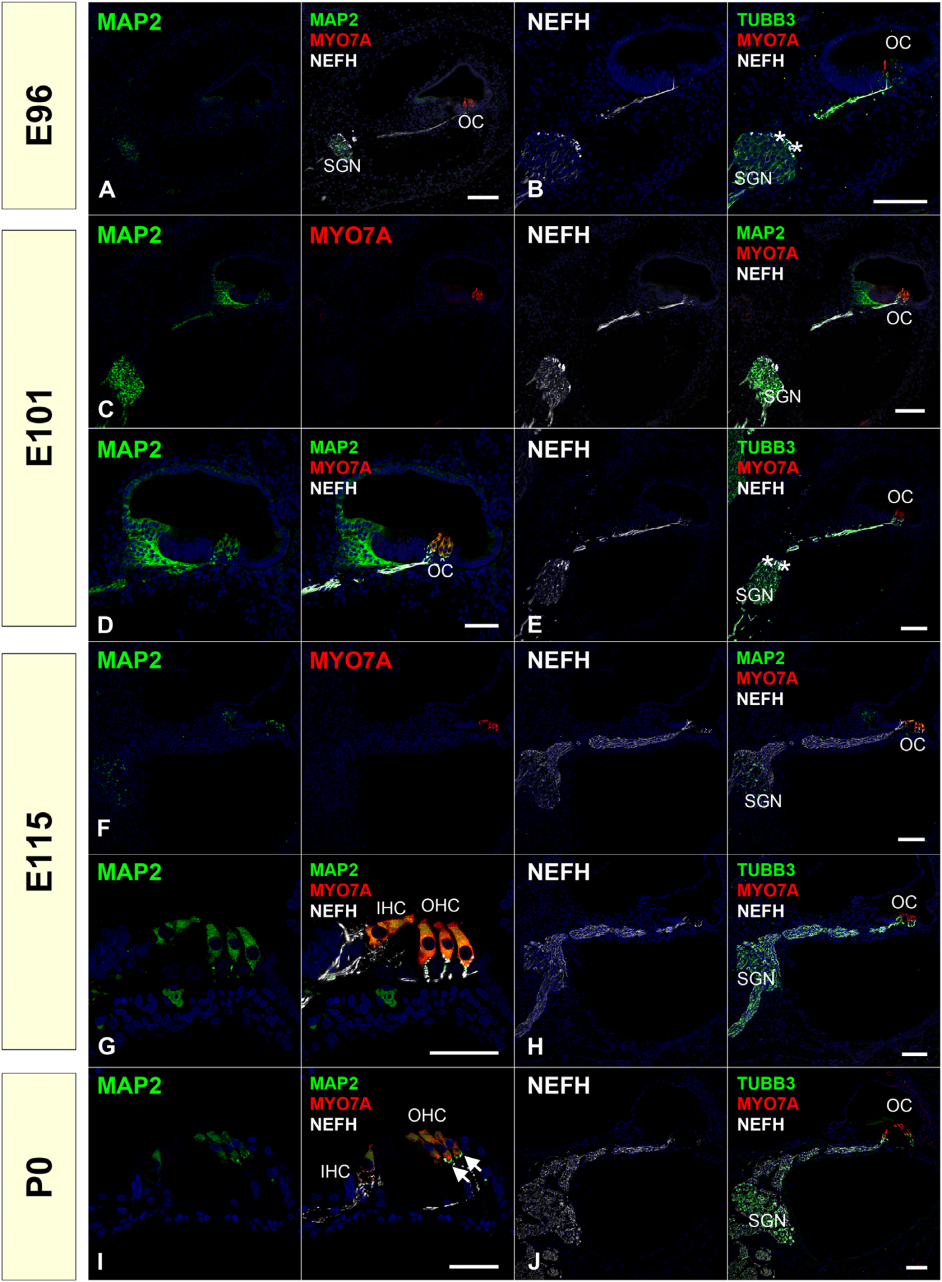
Changes of expression patterns of MAP2, TUBB3, and NEFH during development. (a) At E96, weak MAP2 expression can be detected in neurons within the spiral ganglion. (b) At E96, NEFH expression is relatively higher in the IGSB (asterisk in b). (c) and (d) Most intensive MAP2 expression can be observed at E101. MAP2 expression can be seen in spiral ganglion neurons, modiolus half of sensory epithelium, and inner and outer hair cells at E101. (e) At E101, TUBB3 and NEFH expression can be detected at spiral ganglion. At E101, NEFH expression is relatively still higher in the IGSB (asterisk in e). (f) and (g) The expression of MAP2 in spiral ganglion was decreased at E115. The only slight expression can be detected in spiral ganglion neurons. MAP2 expression was observed in both inner hair cells and outer hair cells at this stage. (h) At E115, TUBB3 and NEFH expression can be detected at spiral ganglion. (i) MAP2 expression in inner and outer hair cells can still be detected in P0. No expression was detected in the spiral ganglion neurons except for only a slight expression observed in the neurite's tip under the organ of Corti (arrow in i). (j) At P0, TUBB3 and NEFH expression can be detected at spiral ganglion. No expression of TUBB3 and NEFH could not be detected in both hair cells in the stages we used in this study. The nuclei were counterstained with Hoechst (blue). Scale bar: 100 μm in (a)–(c), (e), (f), (h), and (j), 50 μm in (d), (g), and (i). SGN: spiral ganglion neurons, OC: organ of Corti, IHC: inner hair cells, OHC: outer hair cells

MAP2 is a protein that belongs to the microtubule‐associated protein family. The proteins of this family are thought to be involved in microtubule assembly, which is an essential step in neuritogenesis. We observed only slight MAP2 expression at E96; however, its expression was detected more broadly at E101, including in the development of the spiral limbus. After that, at E115 and P0, its expression was restricted to both the inner and outer hair cells and neuronal terminals under the hair cells (Figure 2). Compared to TUBB3 (tubulin β 3 class III) expression (Figure 2), a β‐tubulin protein that forms a dimer with α‐tubulin and acts as a structural component of microtubules, MAP2 expression showed dynamic changes in expression patterns. The TUBB3 expression was consistently observed in the spiral ganglion neurons and could not be detected in either of the hair cell stages used in this study.

The MAP2 expression in the cochlea has been reported in guinea pigs (Slepecky & Ulfendahl, 1992), rats (Hafidi et al., 1992), and humans (Anniko & Arnold, 1995). Hafidi et al. reported the expression pattern of MAP2 in the developing cochlea of rats. They showed that MAP2 labeling occurred in the perikarya and neurites of both the developing spiral ganglion and developing neurites. They also reported that no MAP2 immunolabeling was observed in the IGSB, and MAP2 was not present in efferent axons of the central nervus system that project to the cochlea (Hafidi et al., 1992). The expression of MAP2 in hair cells in adults has been reported in both rodents (Ladrech & Lenoir, 2002; Slepecky & Ulfendahl, 1992) and humans (Anniko & Arnold, 1995). However, changes in the MAP2 expression in the human fetus have not been reported. In this study, we showed the dynamic expression changes of MAP2 in the nonhuman primate fetus. Similar to the rodent model, MAP2 expression was not observed in the IGSB of the common marmoset. Our results comparing GAP43 and MAP2 expression patterns in early innervating neurons indicate that GAP43 is preferable for detecting more immature neurites in the common marmoset.

### 2.2 Changes in the expression pattern of neurofilaments during development

Next, we investigated the cytoskeletal structural composition of the developing spiral ganglion neurons. Intermediate filaments are cytoskeletal structural components found in vertebrate cells. There are five principal neuronal‐specific intermediate filaments: light, medium, and heavy molecular mass neurofilament triplet proteins (NF‐L (neurofilament light), NF‐M (neurofilament medium), and NF‐H (neurofilament heavy)), INA (α‐internexin), and PRPH (peripherin). Mature filaments are composed of several combinations of these five subunits. In this study, we first examined the expression patterns of NEFH, NEHM, and NEFL.

*NEFH* encodes a neurofilament heavy polypeptide (NF‐H; NF 200). NEFH expression was observed at E96. The expression of NEFH was continuously observed in spiral ganglion neurons during our observation period from E96 to P0 (Figure 2).

**FIGURE 3:**
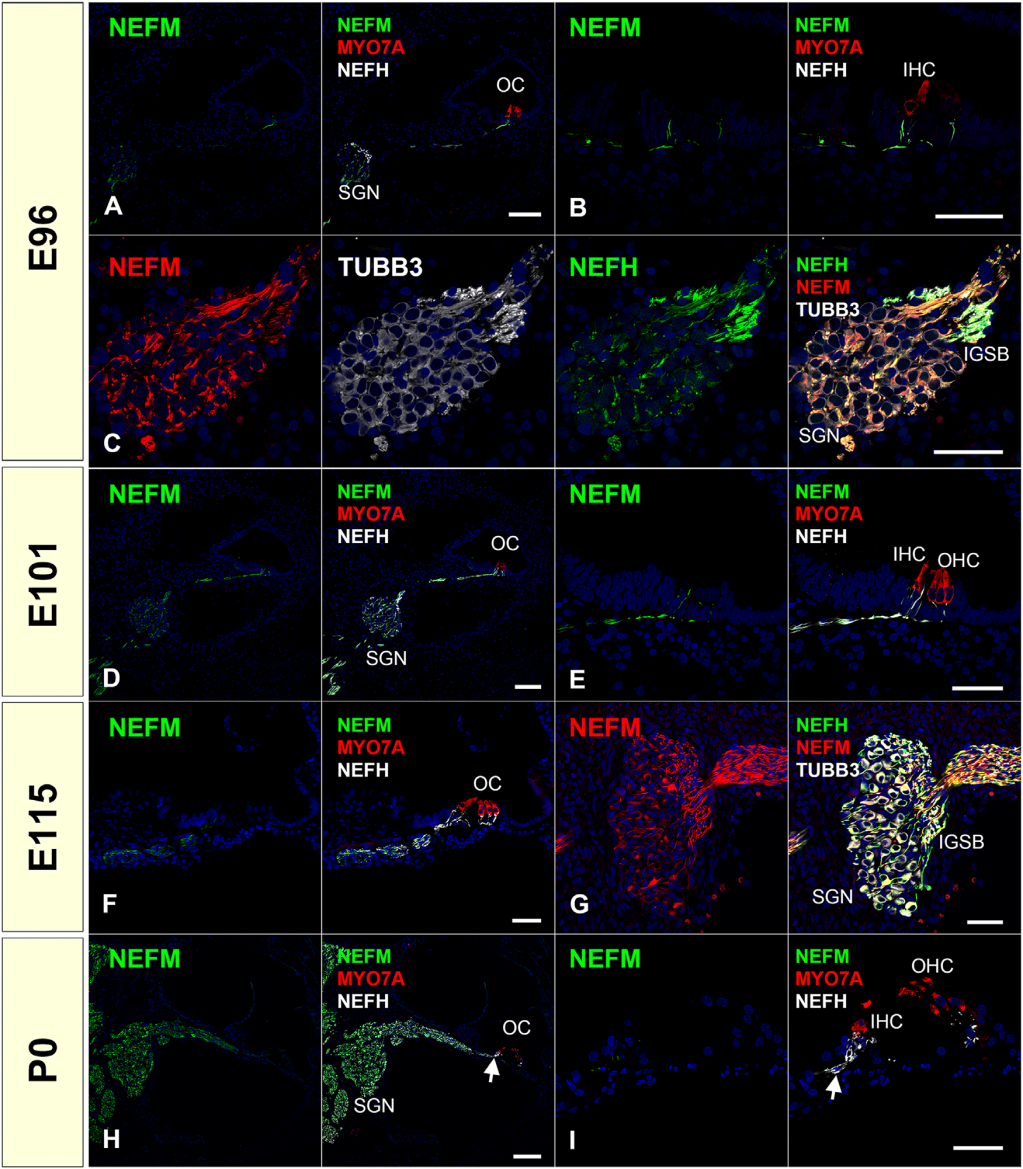
Changes of expression patterns of NEFM during development. (a)‐(c) NEFM expression was detected in spiral ganglion neurons at E96. Comparing with the expression of NEFH, NEFM expression was relatively weak in IGSB (c). (d) and (e) NEFM expression continued to be observed in the spiral ganglion neurons of E101. (f) and (g) NEFM expression continued to be observed in the spiral ganglion neurons of E115. (h) and (i) While NEFM expression is still kept in the spiral ganglion neuron, its expression in more distal neurons from Habenula perforata was diminished (arrow in h and i). Nuclei were counterstained with Hoechst stain (blue). Scale bar: 100 μm in (a), (d) and (h), 50 μm in (b), (c), (e)–(g), and (i). SGN, spiral ganglion neurons; OC, organ of Corti; IHC, inner hair cells; OHC, outer hair cells; IGSB, intraganglionic spiral bundle

*NEFM* encodes neurofilament medium polypeptide (NF‐M, NF150, and NF160). In common marmosets, we noted coexpression of NEFM and NEFH during the observed stages (Figure 3). However, we observed decreased expression of NEFM in the organ of Corti after E101, and no expression of NEFM in the distal neurons of Habenula perforata, in which NEFH expression was detected, at E115 and P0 (Figure 3).

**FIGURE 4:**
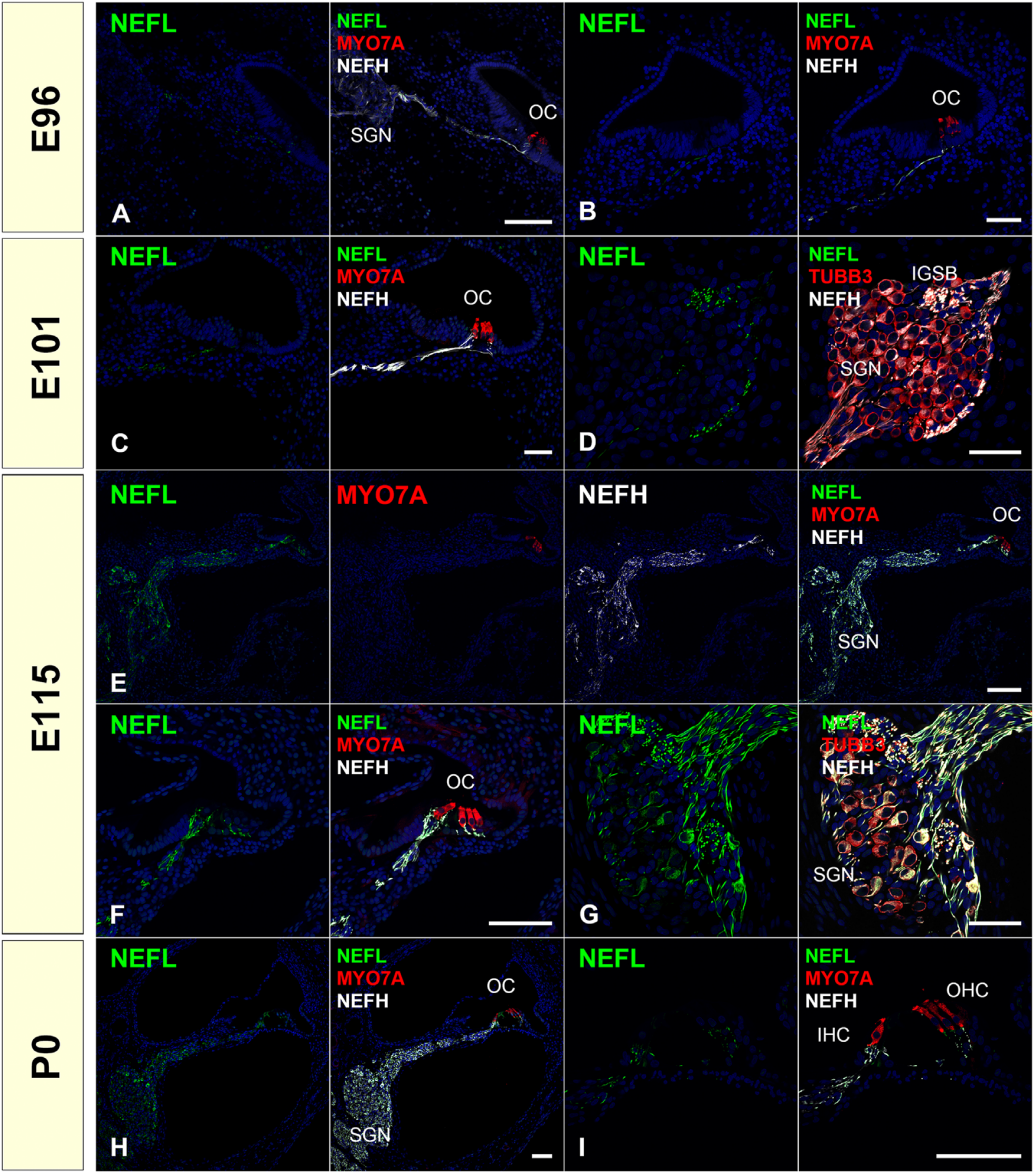
Changes of expression patterns of NEFL during development. (a) and (b) NEFL expression was not detected at E96. (c) and (d) NEFL expression was detected in IGSB at E101, while no expression was observed in the perikaryon of spiral ganglion neurons. (e)‐(g) NEFL expression comes to be spread over the full length of spiral ganglion neurons at E115. NEFL expression can be observed in neurites innervated to hair cells (f). (h) and (i) At P0, NEFL expression was kept in the spiral ganglion neuron. NEFL expression is wholly matched with NEFH expression at P0. Nuclei were counterstained with Hoechst stain (blue). Scale bar: 50 μm in (b)–(d) and (g), 100 μm in (a), (e), (f), (h), and (i). SGN: spiral ganglion neurons, OC: organ of Corti, IHC, inner hair cells; OHC, outer hair cells; IGSB: intraganglionic spiral bundle

*NEFL*, which encodes neurofilament light polypeptide (NF‐L, NF 68), showed a characteristic expression pattern in common marmosets compared to NEFM and NEFH (Figure 4). We detected NEFL expression in the IGSB at E101, and its expression increased and spread as development proceeded. In the P0 cochlea, its expression was detected in all NEFH‐positive neurites, including more distal NEFM‐negative from Habenula perforata (Figure 4h,i).

**FIGURE 5:**
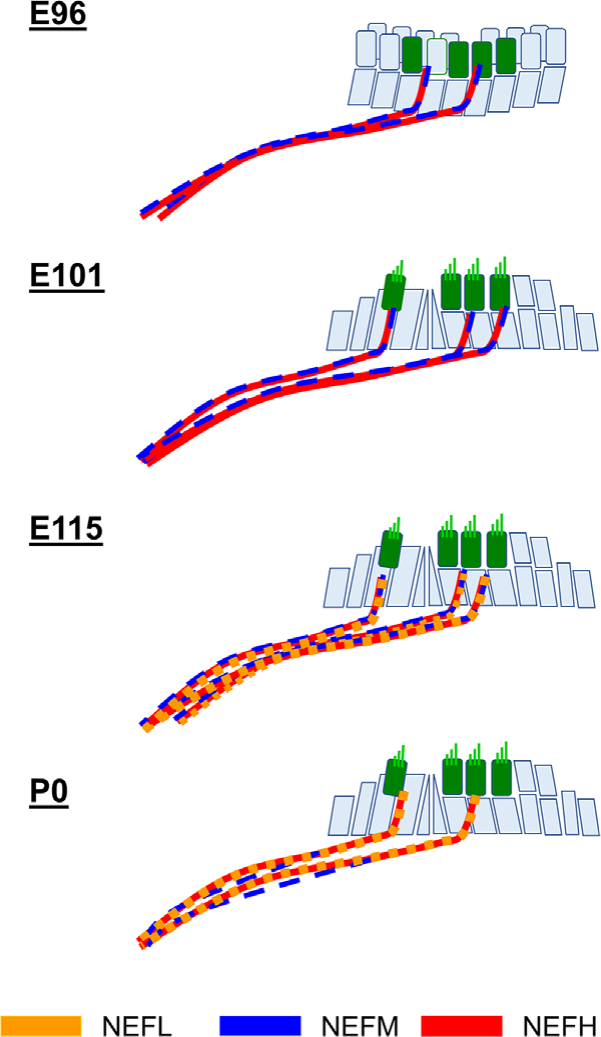
Schematic diagram of developmental changes of neurofilament composition in the common marmoset. Changes in the neurofilament expression patterns are shown. In the early stages, NEFM and NEFH were dominant. NEFL expression spread in the late stages

The composition or assembly of neurofilament triplets (NF‐L, NF‐M, and NF‐H) can differ depending on the type of neuron (Scott et al., 1985; Tohyama et al., 1991), developmental stages (Schlaepfer & Bruce, 1990; Tohyama et al., 1991), or species (Carter et al., 1998; Tsang et al., 2000). Previously, Nishizaki and Anniko (1995) and Tonnaer et al. (2010) reported that in the rat cochlea, the expression of NEFL and NEFM is followed by NEFH expression. However, our observations showed that in the common marmoset, the expression of NEFL was observed in relatively later stages (Figure 5), compared to the expression pattern previously reported in the human spinal cord (Tohyama et al., 1991).

Reduced neurofilament expression has been reported as a general phenomenon after axonal injury in both the peripheral and central nervous systems (Hoffman & Cleveland, 1988; Hoffman et al., 1993). The reemergence of the neurofilament subunit in a temporal sequence, as observed in the developmental process of the human spinal cord, has also been reported (Zhao & Szaro, 1995). Therefore, we postulate that the temporal sequences of neurofilament subunits expression we observed in the developing primate cochlea (Figure 5) could also be useful for future studies on axonal regeneration of spiral ganglion neurons in humans.

### 2.3 Myelination of spiral ganglion neurons

The rapid transduction of electrical neuronal signals along with peripheral neurites can be achieved by the myelination of neurites. In the afferent neurons in the cochlea, type I spiral ganglion neurons have myelinated fibers, whereas type II spiral ganglion neurons lack the myelin sheath. In the efferent system, neurons that innervate outer hair cells (medial olivocochlear efferent) have myelinated fibers, while neurons that innervate inner hair cells (lateral olivocochlear efferent) lack them. Myelination of spiral ganglion neurons is essential for the ability of humans to hear sound. Delayed myelination in infants causes auditory neuropathy (Psarommatis et al., 2017). Moreover, the relationship between the decrease in myelination in aged patients and age‐related hearing loss has been reported (Felder & Schrott‐Fischer, 1995; Xing et al., 2012).

**FIGURE 6:**
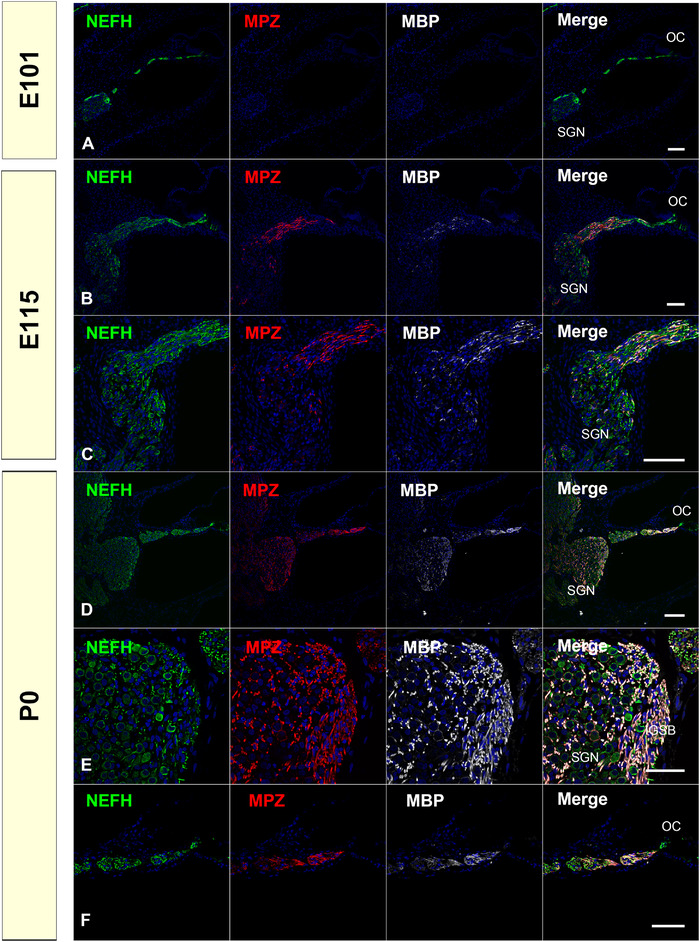
Changes of expression patterns of MPZ and MBP during development. (a) No expression of MPZ and MBP was observed in the cochlea at E96 (b) and (c). At E115, MPZ and MBP can be detected in the osseous spiral lamina, while their expressions are still relatively weaker in spiral ganglion neurons. (d)‐(f) At P0, MPZ and MBP expression can be observed in the spiral ganglion neuron and medial neurite from Habenula perforata. Most of the perikarya of the spiral ganglion neurons were not surrounded by MPZ‐MBP‐positive myelin sheath (d). No expression was observed in the organ of Corti (f). Nuclei were counterstained with Hoechst stain (blue). Scale bar: 50 μm in (e), 100 μm in (a)–(d). SGN, spiral ganglion neurons; OC, organ of Corti; IGSB, intraganglionic spiral bundle.

Myelin protein zero (MPZ) is a membrane glycoprotein, which is a major structural component of the myelin sheath in the peripheral nervous system. *MBP*, which encodes the myelin basic protein (MBP), is another essential gene for myelination. In common marmosets, the expression of MPZ and MBP can be detected synchronously around E115. At P0, we observed MPZ and MBP expression in the spiral ganglion neurons and medial neurites from Habenula perforata (Figure 6).

The time course of myelination in the cochlea has been reported in several species (Moore & Linthicum, 2001; R. Romand & M. R. Romand, 1985; Roth & Bruns, 1993; J. Wang et al., 2013). In mice, MPZ was detected in the soma region of spiral ganglion neurons as early as P0, and the first expression of MPZ in peripheral spiral ganglion neurons was observed in P5. Its expression was increased from P7 to P10 and finally ended at P14 (J. Wang et al., 2013). In rats, the first myelination of spiral ganglion neurons was detected on P6 (R. Romand & M. R. Romand, 1985). In humans, by GW15, Schwann cells are seen in the modiolus and along the spiral lamina. Then, by the GW24, light myelin sheaths extend up to the glial junction (Moore & Linthicum, 2001). In this report, we observed the myelination of peripheral spiral ganglion neurons in the common marmoset at E115 (equivalent to P9 of mouse and GW20 in humans about cochlear development; Hosoya et al., 2021) (Figure 6b,c). Compared to rodents and previous human reports, the myelination process in the common marmoset is similar to that in rodents and relatively slower than in humans. However, morphologically, most of the spiral ganglion neurons in P0 lack MPZ or MBP expression around their perikaryon, whereas in mice the perikaryon is surrounded by a myelin sheath (Glueckert et al., 2005; Ota & Kimura, 1980; M. R. Romand & R. Romand, 1987). This indicates that, anatomically, the myelination pattern of the common marmoset is more similar to that of humans than rodents.

It has been reported that the timing of the start of myelination correlates with the loss of GAP43 expression (Bartolome et al., 2002). We observed this preserved inverse pattern of myelination and GAP43 expression in the developing cochlea of the common marmoset, while MPZ expression started from the soma region in mice (J. Wang et al., 2013) and from the more peripheral region in the common marmoset (Figures 1 and 6).

### 2.4 Expression patterns of neurotrophins and their receptors in developing primate cochlea

The developmental process of inner ear cells, accompanied by their specification and characterization, is tightly controlled by several factors, depending on the developmental stage and cell type. Neurotrophins are crucial elements for neuronal cells for their elongation toward the cochlea and survival after their innervations. Between the neurotrophins, the brain‐derived neurotrophic factor (BDNF) and neurotrophin 3 (NTF3) are essential factors for cochlear neuronal development. BDNF acts on specific neurons of the central and peripheral nervous systems, supporting the survival of existing neurons and encouraging the growth and differentiation of new neurons and synapses. NTF3 contributes to neuronal differentiation, survival, and axonal outgrowth. In the postnatal mouse cochlea, NTF3 is necessary for the formation and maintenance of hair cell ribbon synapses (Wan et al., 2014).

**FIGURE 7:**
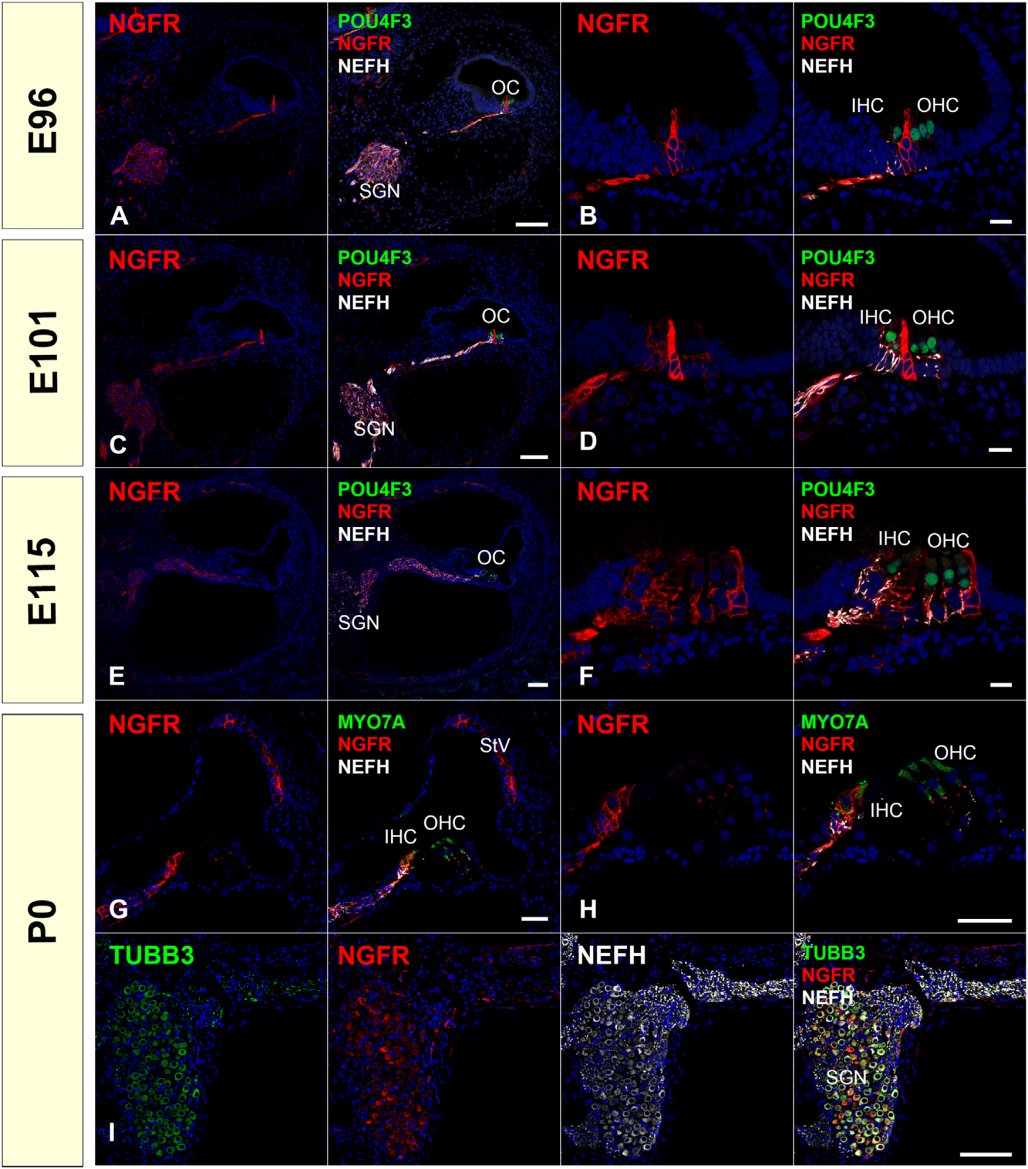
Changes of expression patterns of NGFR during development. (a) and (b) At E96, NGFR expression was detected in the spiral ganglion neurons. Expression of NGFR was also seen in cells between the POU4F3 positive inner hair cells and outer hair cell, which is thought to be future pillar cells. (c) and (d) At E101, NGFR expression was still observed in spiral ganglion neurons and supporting cells. Relatively high expression was observed in the pillar cells (d). (e) and (f) At E115, expression of NGFR is observed in the spiral ganglion neurons and supporting cells. In the organ of Corti, expression of NGFR was detected in inner border cells, inner pharyngeal cells, inner and outer pillar cells, and Deiters' cells (f). (g)–(i) At P0, NGFR expression was detected in inner border cells and inner pharyngeal cells (h) and spiral ganglion neurons (i). At this stage, NGFR expression was also observed in stria vascularis (g). The nuclei were counterstained with Hoechst (blue). Scale bar: 100 μm in (a), (c), (e) and (i), 50 μm in (g) and (h), 20 μm in (b), (d), and (f). SGN: spiral ganglion neurons, OC: organ of Corti, IHC: inner hair cells, OHC: outer hair cells, StV: stria vascularis

Neurotrophins bind to two different receptor types. NGFR (nerve growth factor receptor, P75NTR) is one of the two receptor types of neurotrophins, and NGFR binds all neurotrophins with low affinity. NGFR signaling contributes to promoting apoptosis, inhibits neurite growth, and depresses synaptic strength (Teng et al., 2010). We observed NGFR in the inner ear; NGFR expression has been reported in spiral ganglion neurons (Despres et al., 1991; Gestwa et al., 1999; von Bartheld et al., 1991). A transient expression of NGFR in pillar cells has also been reported in developmental rodent cochlea (Despres et al., 1991; Gestwa et al., 1999; von Bartheld et al., 1991). However, no immunoreactivity of the stria vascularis or hair cells was observed (Despres et al., 1991). In adult rodents, it was reported that NGFR was expressed in the majority of the neurons in the spiral ganglion as well as the pillar and supporting cells around the outer hair cells (Schulze et al., 2020). In humans, a broad expression of NGFR is observed in the adult inner ear in neurons, Schwann and satellite cells in the spiral ganglion, as well as in hair cells and supporting cells in the organ of Corti (Johnson Chacko et al., 2017; Liu et al., 2012), and the expression of NGFR in the spiral ganglion in early phase fetuses (GW11) has also been reported (Johnson Chacko et al., 2017). Comparing the above‐mentioned observations in various species, transient expression in the pillar cells and expression in spiral ganglion neurons of NGFR seems well preserved between the species (Figure 7). However, NGFR expression observed in inner hair cells and stria vascularis has not been reported in species other than the common marmoset, including the human fetus. There have been no detailed reports on late‐phase expression patterns of NGFR in the human fetus. Therefore, future investigations should compare our observations in common marmosets with observations in human fetuses.

Another type of neurotrophin receptor is the NTRK (Trk) receptor. The NTRK (neurotrophic receptor tyrosine kinase) family receptor protein‐‐tyrosine kinases are high‐affinity receptors for neurotrophins and consist of three members: NTRK1 (TrkA), NTRK2 (TrkB), and NTRK3 (TrkC). NTRK1, NTRK2, and NTRK3 are the high‐affinity receptors for NGF, BDNF and NTF4 (neurotrophin 4), and NTF3, respectively. Additionally, NTF3 binds with a lower affinity for both NTRK1 and NTRK2. Signaling through these receptor protein‐‐tyrosine kinases is generally hypertrophic and increases neuronal metabolism, specifically promoting neuronal survival, stimulating neurite growth, promoting synaptogenesis, and potentiating synaptic strength (E. J. Huang & Reichardt, 2003). Studies in mutant mice have revealed that loss of BDNF and its receptor NTRK2 impairs innervation along the tonotopic axis of the cochlea, while the deletion of NTF3 and its receptor NTRK3 resulted in the loss of innervation of the spiral ganglion along with the basal turn of the cochlea (Fritzsch et al., 1997). Furthermore, in double knockout mice lacking BDNF and NTF3 or NBTRK2 and NTRK3, complete loss of afferent innervation of the inner ear was observed (Ernfors et al., 1995; Silos‐Santiago et al., 1997).

**FIGURE 8:**
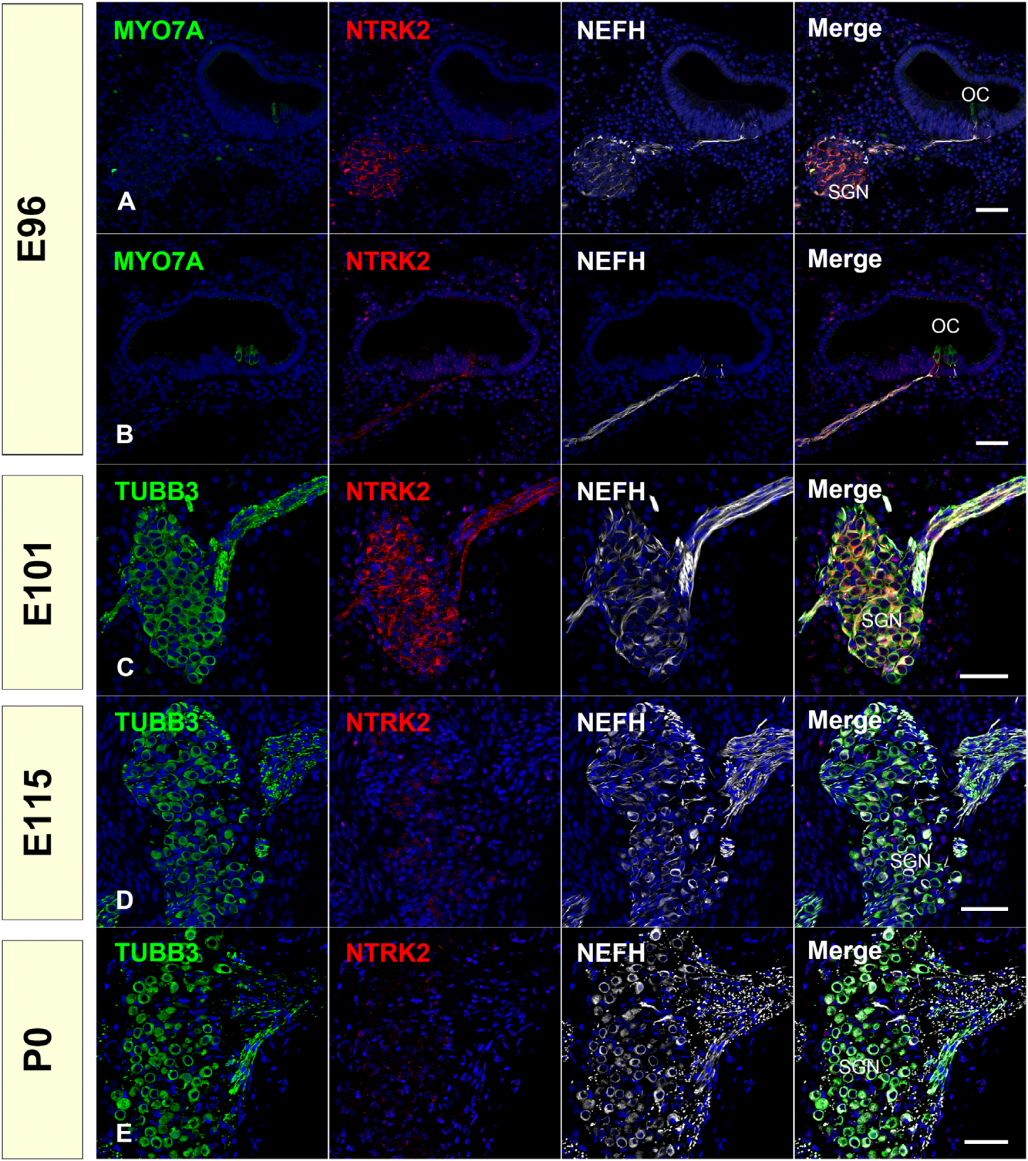
Changes of expression patterns of NTRK2 during development. (a) and (b) At E96, expression of NTRKs can be observed in immature spiral ganglion neurons and extending neurites. (c) At E101, NTRK2 expression was still detected in the spiral ganglion neuron (d) and (e). No expression of NTRK2 was observed at E115 and P0. The nuclei were counterstained with Hoechst (blue). Scale bar: 50 μm. SGN: spiral ganglion neurons, OC: organ of Corti

NTRK2 is a high‐affinity receptor of BDNF. In rodents, it has been reported that NTRK2 expression has been observed in the spiral ganglion until E16.5, whereas its expression was not detected in P0 (Schecterson & Bothwell, 1994). In contrast, in adult humans, expression of NTRK2 in type I and type II spiral ganglion neurons and in the pillar cells of the organ of Corti has been reported (Liu et al., 2011). In human cochlear development, NTRK2 expression has been reported in spiral ganglion neurons (Johnson Chacko et al., 2017). We observed NTRK2 expression only at E96 and E101, but not at E115 and P0. This indicates that the expression of NTRK2 in the cochlea in the common marmoset is more similar to that in rodents than in humans (Figure 8).

**FIGURE 9:**
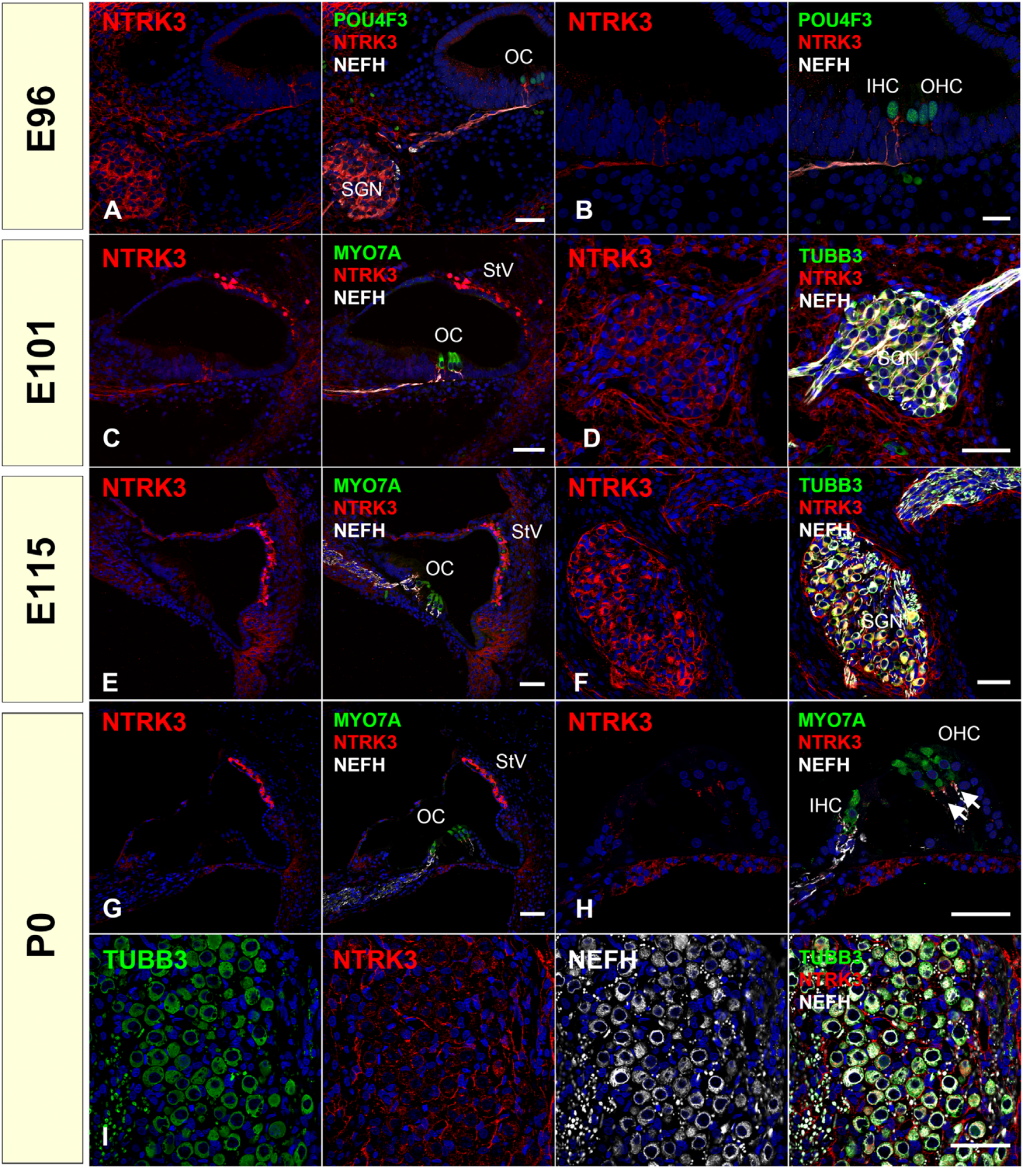
Changes of expression patterns of NTRK3 during development. (a) and (b) At E96, NTRK3 expression was detected in spiral ganglion neurons and nonepithelial connective tissues surrounding spiral ganglion neurons. NTRK3 expression was also observed in neurites in the sensory epithelium (b). (c) and (d) At E101, NTRK3 was observed in spiral ganglion neurons, nonepithelial connective tissues surrounding spiral ganglion neurons, and stria vascularis. At this point, weak NTRK3 expression can also be detected in lateral wall fibrocytes. (e) and (f) At E115, expression of NTRK3 was observed in spiral ganglion neurons and their surrounding connective tissues and stria vascularis. At this point, weak NTRK3 expression can also be detected in lateral wall fibrocytes. (g)‐(i) At P0, NTRK3 expression in spiral ganglion neurons was diminished except for the neuronal terminus under the hair cells (arrow in h). At this point, no NTRK3 expression was detected in the perikaryon of spiral ganglion neurons, while its expression was observed in the surrounding tissue of them (i). At P0, NTRK3 expression was also observed in the intermediate cells of the stria vascularis (g). The nuclei were counterstained with Hoechst (blue). Scale bar: 50 μm in (a) and (c)–(i), 20 μm in (b). SGN: spiral ganglion neurons, OC: organ of Corti, IHC: inner hair cells, OHC: outer hair cells, StV: stria vascularis

NTRK3 is a high‐affinity receptor for NTF3. We observed a broader expression pattern for NTRK3 than NTRK2 in the common marmoset. We detected NTRK3 expression in the spiral ganglion neurons and neurite and nonepithelial connective tissues surrounding the spiral ganglion neurons. NTRK3 expression was also observed in the stria vascularis at E115 and P0 (Figure 9). In rodents, NTRK3 expression has been reported in the prenatal and postnatal spiral ganglion (Ylikoski et al., 1993) and stria vascularis (Gestwa et al., 1999). Thus, the NTRK3 expression pattern was preserved between the common marmosets and rodents. In humans, Johnson Chacko et al. (2017) investigated the expression pattern of NTRK3 in GW9‐12 human fetuses and reported that NTRK3 expression was observed in spiral ganglion neurons. However, no detailed examination of the later stages has been conducted yet.

**FIGURE 10:**
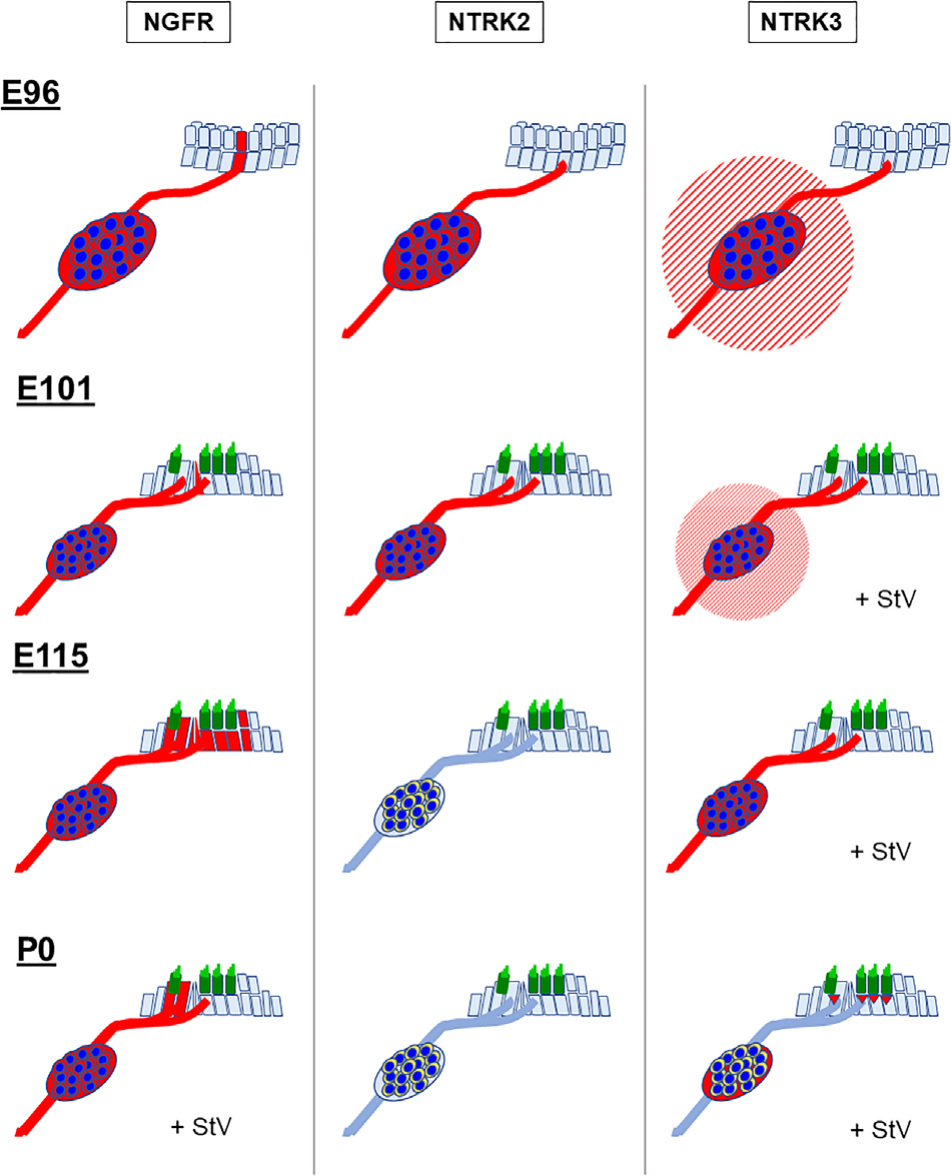
Schematic diagram of developmental changes of neurotrophin receptors in the common marmoset. Changes in the neurotrophin receptor expression patterns are shown. In the early stages, the expression of NGFR, NTRK2, and NTRK3 was detected in spiral ganglion neurons. NTRK2 and NTRK3 expression diminished with development, and only NGFR was observed in the spiral ganglion at P0. StV: stria vascularis

Regarding the expression patterns of the neurotrophin receptor, although most of them are preserved between rodents and primates, several interspecies differences could be observed in our observations. Recently, the administration of neurotrophins to the cochlea has been suggested as a feasible therapy for hearing loss (J. Suzuki et al., 2016). Our results (summarized in Figure 10) indicate that careful recognition of these differences should be used to apply the results obtained from rodents to humans.

### 2.5 Maturation of ribbon synapse

Next, we investigated synaptic formation between hair cells and spiral ganglion neurons. In the cochlea, synaptic conduction between hair cells and spiral ganglion neurons can be characterized by specialized ribbon synapses. This ribbon synapse differs significantly from conventional synapses in other organs in terms of structure, function, and molecular organization. Mature afferent hair cell synapses have a synaptic ribbon that connects the monolayers of synaptic vesicles with presynaptic membranes in response to changes in membrane potential mediated by Ca^2+^ channels. Finally, synaptic vesicle docking into the presynaptic membrane occurs, causing a rapid release of neurotransmitters. Using this unique method, hair cells can transmit very fast, accurate, and sustained neurotransmission to spiral ganglion neurons. Therefore, the developmental maturation of this unique ribbon synapse and its associated molecular structures is essential for hearing. Similar ribbon synapses are also found in retinal photoreceptor cells, vestibular organ receptors, retinal bipolar cells, and pinealocytes. In cochlear hair cells, RIBEYE and CACNA1D (calcium voltage‐gated channel subunit alpha1D, Cav1.3) are known to be significant components or essential interacting proteins of ribbon synapses.

**FIGURE 11:**
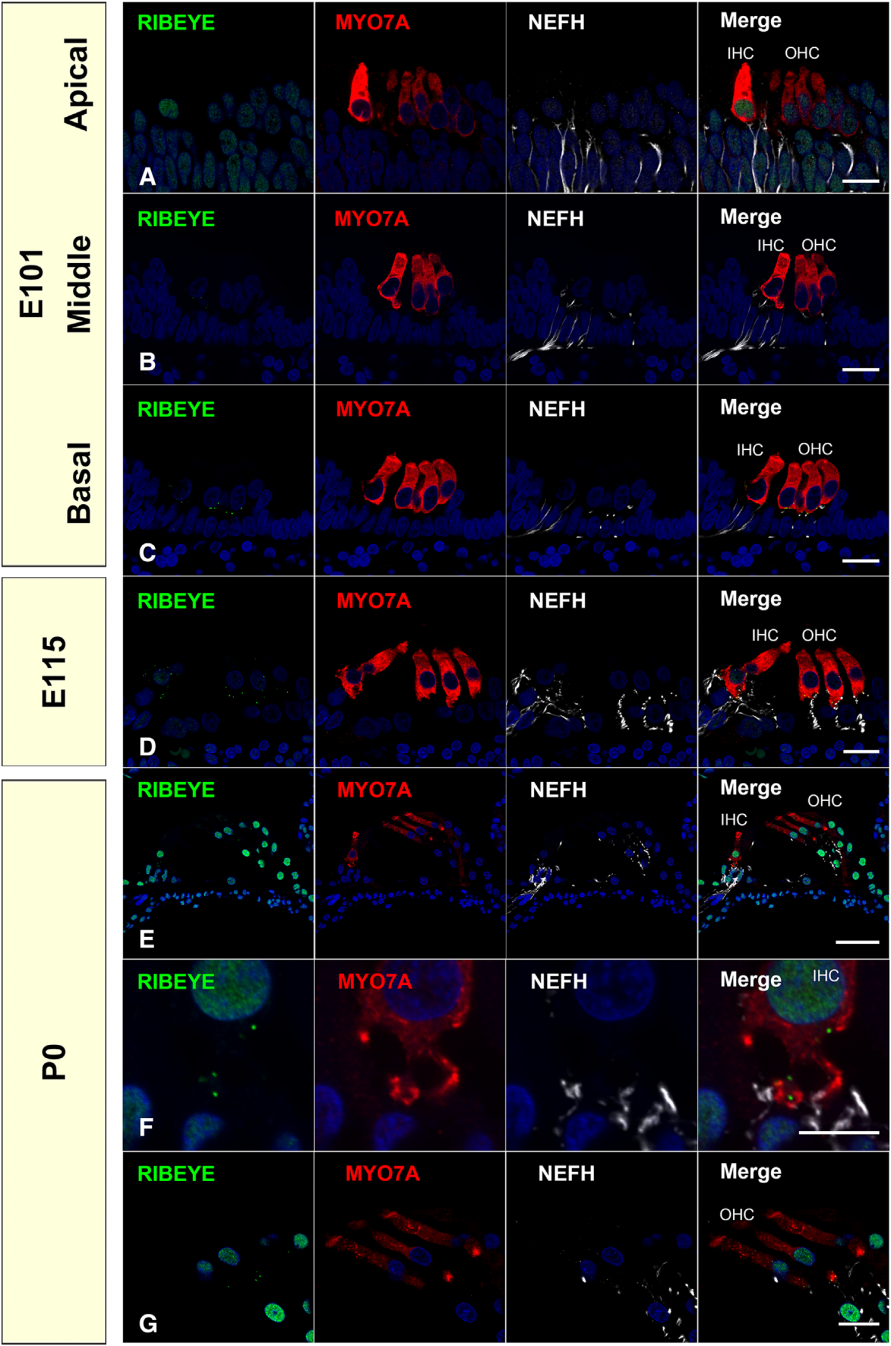
Synaptic formation of the common marmoset in development: expression patterns of RIBEYE. (a)–(c) Expression of RIBEYE was firstly detected in the middle and basal turn of E101 (b) and (c). No RIBEYE expression can be detected in apical turns at this timing (a). (d) At E115, RIBEYE expression was detected on the relatively top side in hair cells. (e)–(g) At P0, RIBEYE expression was restricted to the bottom of both inner hair cells (f) and outer hair cells (g). The nuclei were counterstained with Hoechst (blue). Scale bar: 20 μm in (a)–(d) and (g), 50 μm in (e), 10 μm in (f). IHC: inner hair cells, OHC: outer hair cells

RIBEYE is a significant component of synaptic ribbons, which is encoded by the *CTBP2* (C‐terminal binding protein 2) gene. The mammalian *CTBP2* gene produces alternative transcripts that encode two distinct proteins. One of these proteins is a transcriptional repressor (CTBP2), while the other is RIBEYE. In the common marmoset, RIBEYE expression was first detected in the middle and basal turns of E101, with the intracellular distribution having matured by P0 (Figure 11).

**FIGURE 12:**
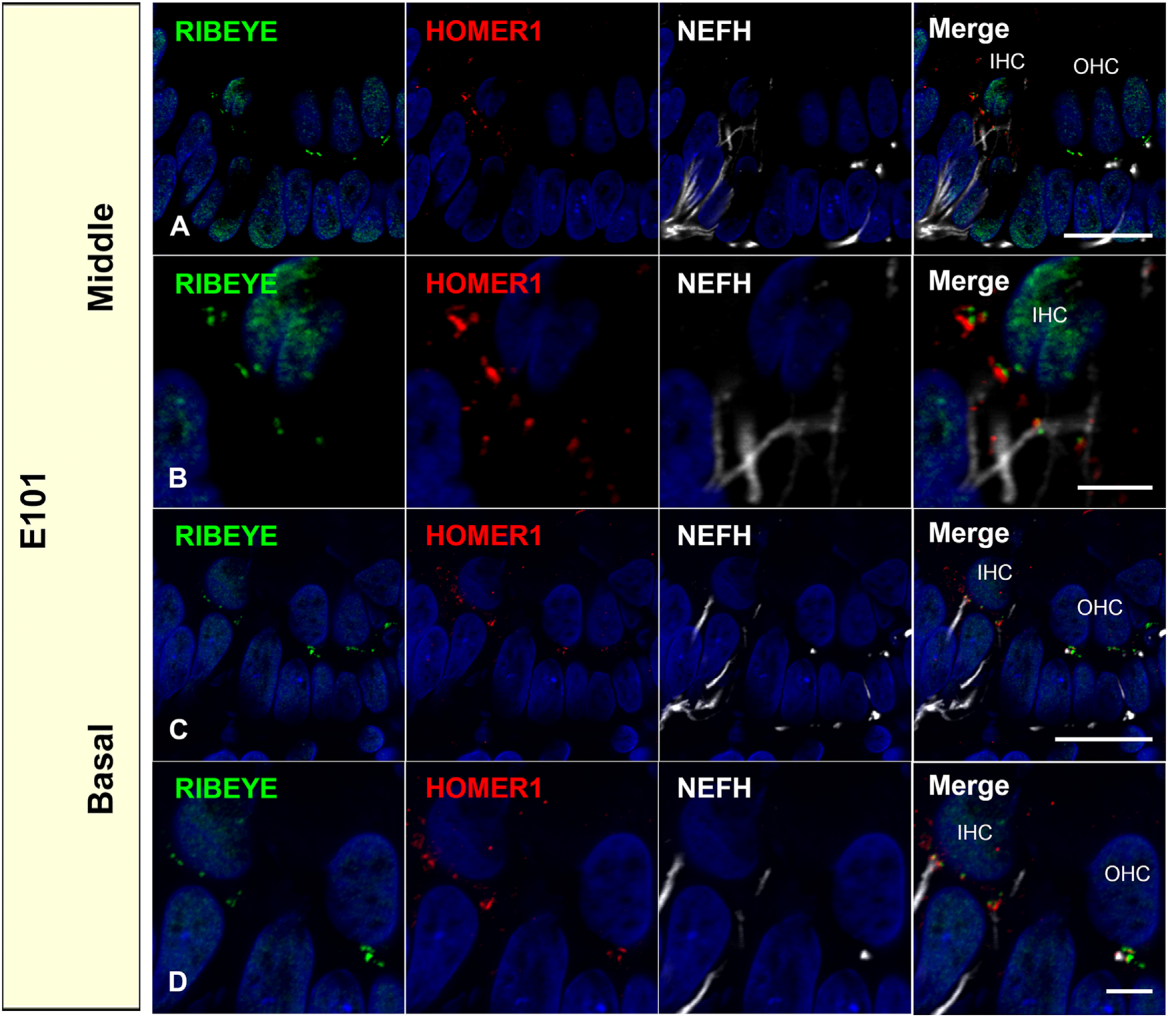
Expression of HOMER1 and RIBEYE at E101 cochlea. Couplings of HOMER1 and RIBEYE were observed at the E101 cochlea in the middle turns (a) and (b) and basal turns (c) and (d). Nuclei were counterstained with Hoechst stain (blue). Scale bar: 20 μm in (a) and (c), 5 μm in (b) and (d). IHC, inner hair cells; OHC, outer hair cells

Next, we investigated the expression of a postsynaptic marker to evaluate the synaptic formation. Homer protein homolog 1 (HOMER1) is a neuronal protein that is concentrated in postsynaptic structures and constitutes a major part of the postsynaptic density and is involved in synaptic plasticity, Ca^2+^ signaling, and neurological disorders. HOMER1 has been used as a postsynaptic marker of spiral ganglion neurons. HOMER1 expression was observed at E101 (Figure 12). The coupling of the expression of HOMER1 and RIBEYE indicates that synaptic connections were formed at the same time as RIBEYE expression.

**FIGURE 13:**
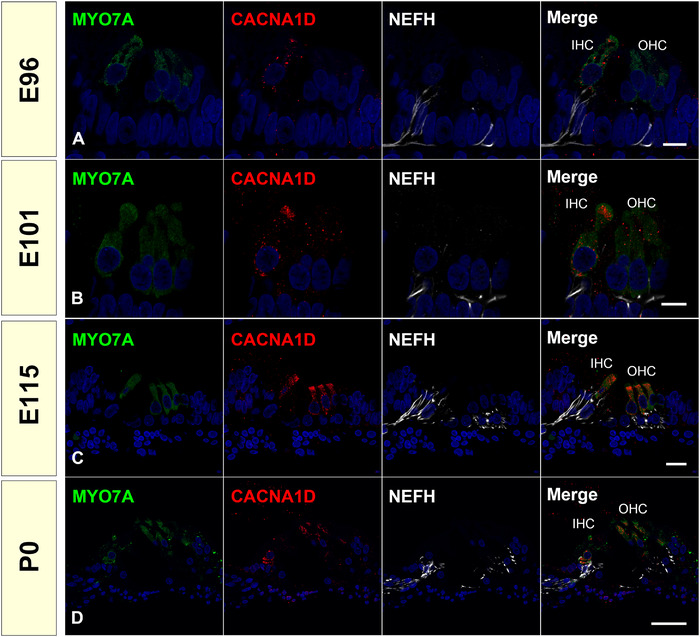
Changes of expression patterns of CACNA1D during development. (a) At E96, CACNA1D expression was detected only in the inner hair cells. (b) At E101, CACNA1D expression was observed in both inner hair cells and outer hair cells. (c) and (d) Continuous expression of CACNA1D in hair cells was detected at E115 and P0. The nuclei were counterstained with Hoechst (blue). Scale bar: 10 μm in (a) and (b), 20 μm in (c), 50 μm in (d). IHC: inner hair cells, OHC: outer hair cells

CACNA1D belongs to the Cav1 family, which forms L‐type Ca^2+^ currents. Voltage‐dependent calcium channels are selectively permeable to Ca^2+^ ions, mediating the movement of these ions in and out of excitable cells. At resting potential, these channels are closed, but when the membrane potential is depolarized, these channels open. The influx of Ca^2+^ ions into the cell can initiate a myriad of Ca^2+^‐dependent processes, including the fusion of synaptic vesicles containing neurotransmitters to the presynaptic membrane of hair cells. Therefore, CACN1D is essential for the development and presynaptic activity of cochlear hair cells (Brandt et al., 2003). In the common marmoset, we detected CACNA1D expression at E96, followed by RIBEYE expression at E101. First, the expression of CACNA1D was dominant in the inner hair cells. However, its expression can be seen in the outer hair cells after E101 (Figure 13).

In mice, RIBEYE puncta were reportedly observed in both inner and outer hair cells as early as E18 (L. C. Huang et al., 2012), and CACNA1D expression was observed in cochlear hair cells from E15 before RIBEYE expression (Engelman et al., 1998). This time course was also well preserved in the common marmoset (Figures 11, 12, 13). In human cochlear development, anatomical observations of synaptic formation have also been reported. Presynaptic specializations are first observed at GW11–12 at the base of inner hair cells and outer hair cells, and the number of synaptic contacts was increasing at GW14 (Lavigne‐Rebillard & Pujol, 1988; Pujol & Lavigne‐Rebillard, 1985). However, no detailed observation of the expression pattern of RIBEYE or HOMER1 has been made in the human fetus. Considering our observation in the common marmoset, in which anatomical innervation of the afferent neuron is followed by expression of RIBEYE expression a few days later, we postulate that human RIBEYE expression would be expected to be observed in GW14‐16. However, a detailed examination of the human fetus is required in future studies to determine the validity of this postulation.

### 2.6 Differentiation of type I and type II neurons

Efferent neurons of the spiral ganglion can be divided into two types: type I and type II neurons. Type I neurons connect to the inner hair cells, whereas type II neurons connect to the outer hair cells. Previously, we reported that the developmental patterning of these two types of neurons was different from that of primates and rodents, observing PRPH expression patterns. Here, we examined other type‐specific marker expression patterns, including ATPase Na+/K+ transporting subunit alpha 3 (ATP1A3), calbindin (CALB1), and calretinin (CALB2).

*ATP1A3* encodes ATP1A3, a membrane‐bound transporter that uses ATP to transport three Na^+^ outwards in exchange for two K^+^ ions into the cell, resulting in a negative transmembrane potential. The expression of ATP1A3 has been reported in type I but not in type II spiral ganglion neurons in rats (McLean et al., 2009).

**FIGURE 14:**
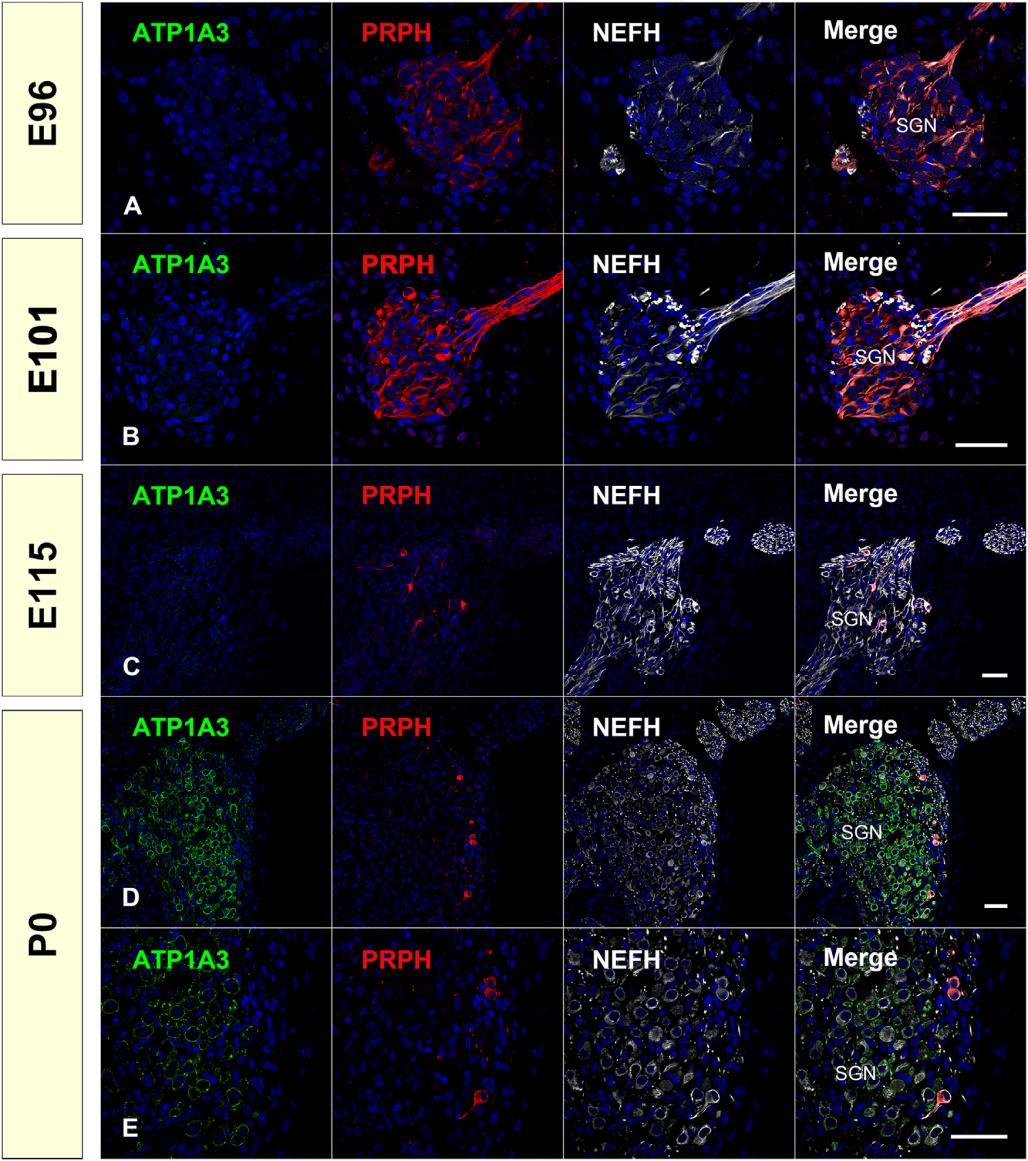
Changes of expression patterns of ATP1A3 and PRPH during development. (A) and (b) At E96 and E101, no ATP1A3 expression was observed in the spiral ganglion neurons, while PRPH expression was observed in most of them. (c) At E115, PRPH expression was restricted into the type II neurons, while no expression of ATP1A3 was observed. (d) and (e) At P0, ATP1A3 expression can be observed in type I neurons, which is PRPH negative. The nuclei were counterstained with Hoechst (blue). Scale bar: 50 μm. SGN: spiral ganglion neurons

In the spiral ganglion of the common marmoset, we did not detect ATP1A3 expression until E115. At P0, ATP1A3 expression was detected in the PRPH‐negative type I spiral ganglion neurons. This result indicates that in the common marmoset, ATP1A3 could be used as a marker of type I spiral ganglion neurons only in the late stages of cochlear development (Figure 14).

**FIGURE 15:**
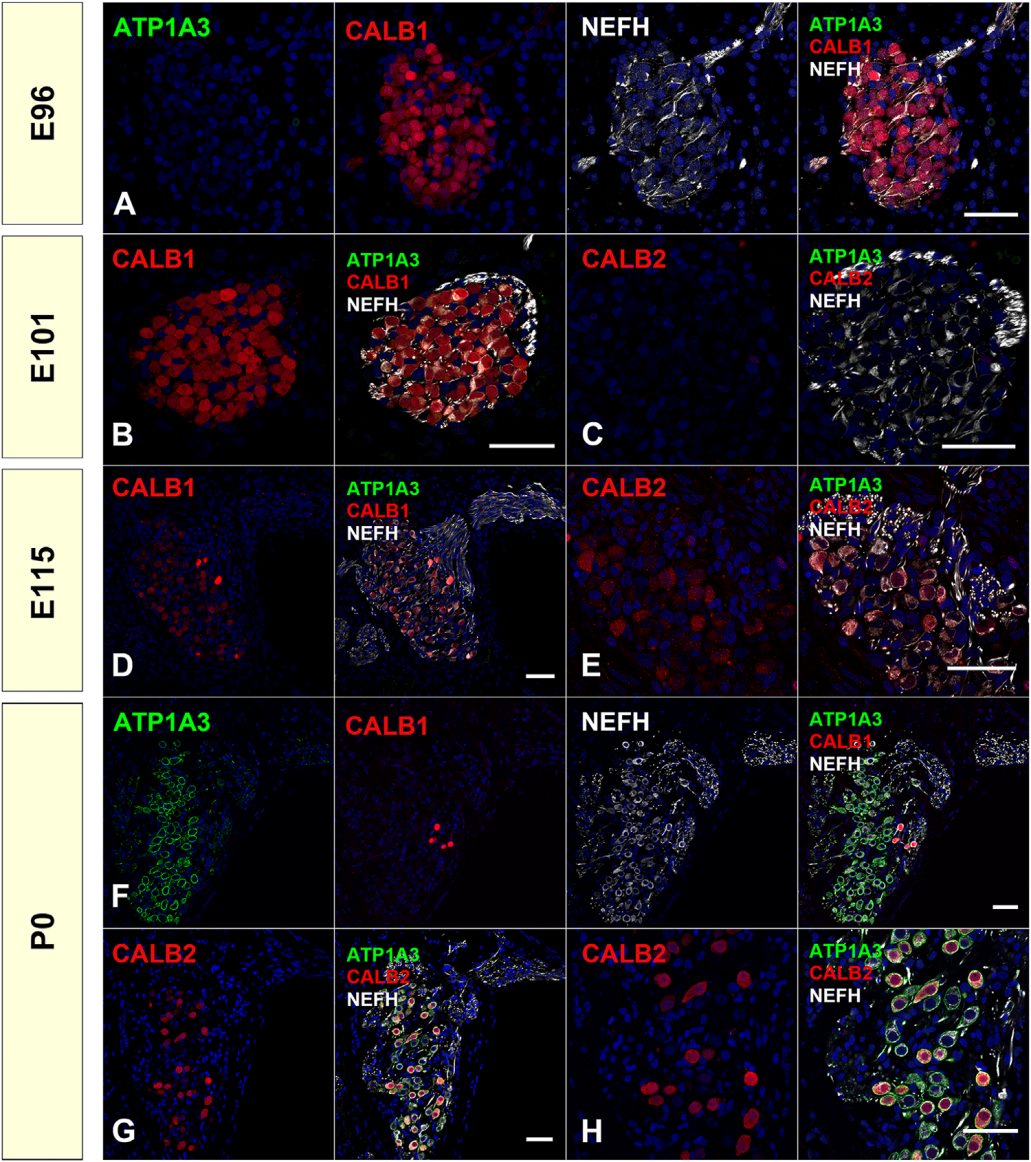
Changes of expression patterns of CALB1 and CALB2 during development. (a) and (b) At E96 and E101, CALB1 expression was broadly observed in the perikaryon of the spiral ganglion neuron. (c) At E101, no CALB2 expression was detected in the spiral ganglion neurons. (d) At E115, the expression of CALB1 comes to be diminished from a part of spiral ganglion neurons. (e) CALB2 expression was observed in a part of the perikaryon of spiral ganglion neurons. (f) At P0, CALB1 expression was detected only in type II neurons (ATP1A3‐negative neurons). (g) and (h) At P0, CALB2 expression was observed in the part of type I neurons (ATP1A3‐positive). The nuclei were counterstained with Hoechst (blue). Scale bar: 50 μm

*CALB1* encodes CALB1, a Ca^2+^‐binding protein involved in regulating mechanoelectrical transduction mechanisms and afferent synaptic neurotransmitter release in the inner ear. *CALB2* encodes another Ca^2+^‐binding protein, CALB2. There is a previous report on the expression pattern of CALB1 and CALB2 in the cochlea of the common marmoset (Spatz & Lohle, 1995). CALB1 is expressed exclusively in the common marmoset spiral ganglion by type II cells, and CALB2 is expressed in a part of type I cells in neonates and adults, while there are no reports about developmental changes in the fetus of the common marmoset. Interestingly, in the retina, large interspecies differences in the localization of both Ca^2+^‐binding proteins have been reported in monkeys, pigs, sheep, rats, cats, pigeons, and salamanders (Pasteels et al., 1990), while the expression pattern was similar between the primates (Roski et al., 2018). In this study, we investigated the changes in the expression patterns of CALB1 and CALB2 in the development of common marmosets (Figure 15).

In the spiral ganglion of the common marmoset, CALB1 expression was detected in all immature perikarya at E96 and E101 (Figure 15a,b). However, after E115, its expression was restricted to several populations, and finally, at P0, its expression was restricted to type II neurons (Figure 15d,f). In the spiral ganglion of the common marmoset, CALB2 expression was first detected at E115. In E115, CALB2 expression was detected in most of the perikaryons of the spiral ganglion neurons. However, at P0, its expression was restricted to a subpopulation of type I neurites (Figure 15g,h).

It has been reported that the distribution of the expression of CALB1, which heterogeneously labels the spiral ganglion, changes with cochlear development (Buckiova & Syka, 2009). In postnatal mice, it was reported that CALB1 expression was observed in both type I and type II neurons, although relatively higher expression of CALB1 was observed in PRPH positive type II neurons compared to type I neurons (Liu & Davis, 2014). However, our results indicated that, while both type I and type II neurons were CALB1 positive, by birth CALB1 expression was restricted to only type II neurons. This observation is in line with previous observations of adult and neonate common marmosets (Spatz & Lohle, 1995). In humans, Dechesne et al. (1988) reported that in a GW10 human fetus, CALB1 expression was observed in most of the spiral ganglion neurons, while its expression in the later stage has not yet been reported. In mice, the expression of CALB2 in the spiral ganglion was observed at E19, P4, and P7, before finally being restricted to type I neurons in adult mice (Dechesne et al., 1994). Recent observations using single‐cell RNA‐seq in mice revealed that *Calb2* expression levels are different between subtypes of type I neurons correlated with their spontaneous firing rate (Shrestha et al., 2018). Our observation of the expression pattern of common marmosets indicated that this expression pattern of CALB2 is well preserved between the species, and the heterogeneity of type I spiral ganglion has already been evident at birth in primates. In contrast, CALB1 expression showed different patterns between rodents and common marmosets.

**FIGURE 16:**
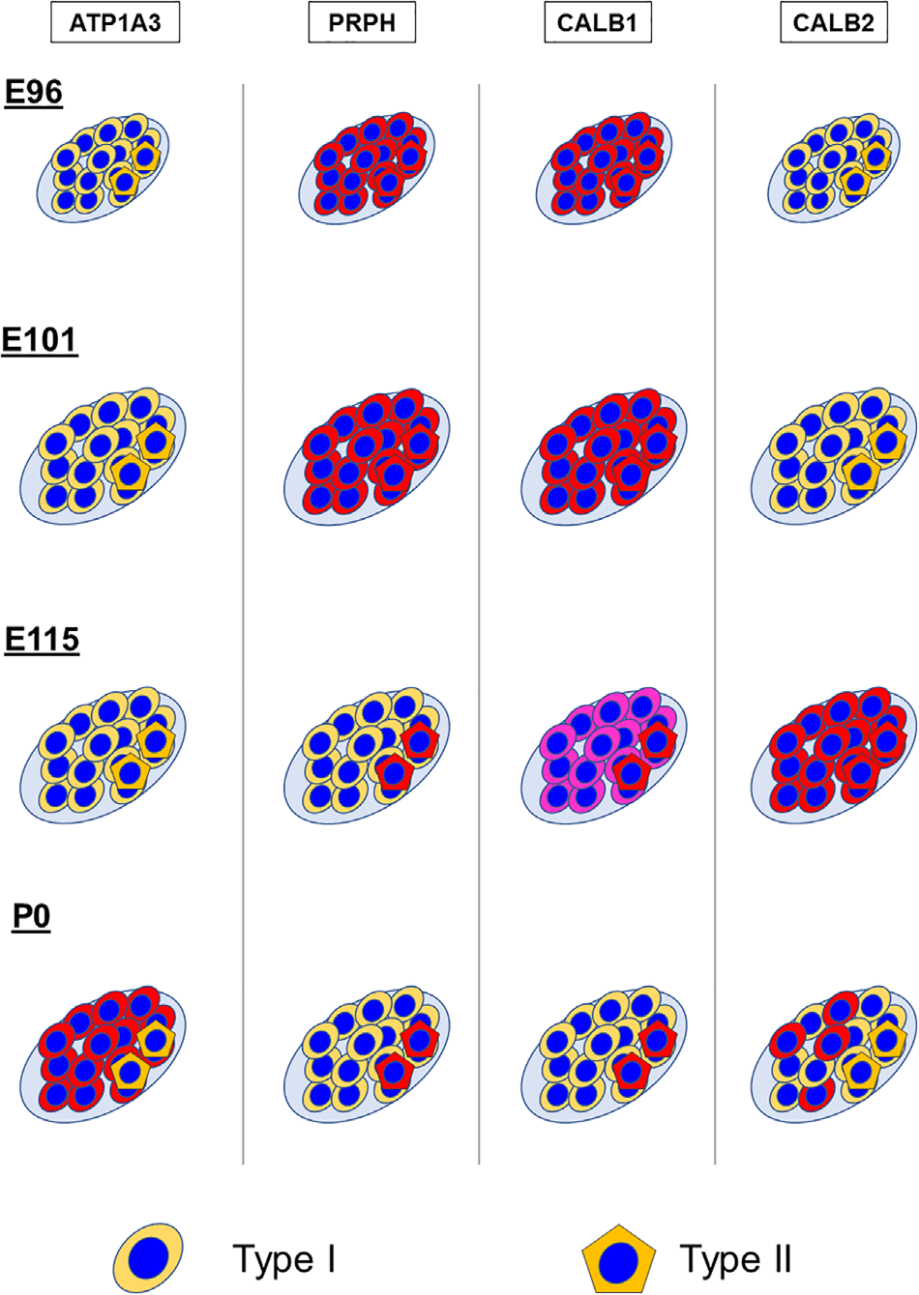
Schematic diagram of delineation of the spiral ganglion neurons during development

In the common marmoset cochlear development, after E115 (equivalent to P9 of the mouse), differences in type I and II spiral ganglion neurons became evident (Figure 16), indicating that their developmental fates may have been determined earlier. The precise timing of neuronal fate specification between type I and type II spiral ganglion neurons has not been determined. In mice, it has been reported that type I and type II spiral ganglion neurons developed from common pools of precursors, and the fates of type I and type II ganglion neurons are determined by E16.5 at the latest in the mouse. These fate decisions are independent of interactions with fully differentiated hair cells (Koundakjian et al., 2007), and any sort of transcriptional cascade associated with the decision remains unknown. Previous reports (Hosoya et al., 2021; Locher et al., 2013) and our results indicate that characteristics of type I and type II spiral ganglion neurons showed clear interspecies differences between rodents and primates; therefore, further research on the primate model is meaningful to examine this phenomenon. A more detailed study examining pre‐E96 (equivalent to E16 in mouse) marmoset tissue is needed to clarify the delineation of primate spiral ganglion neurons.

### 2.7 Efferent nerve innervation and marker expression

Neurons composed of the efferent pathway have distinct developmental steps because of their unique developmental origin; other neuronal cells in the cochlea, including inner and outer hair cells, and type I and type II spiral ganglion neurons (afferent pathway), are developed from common otic placode, while efferent nerves are developed from visceral motor neuron progenitors in rhombomere 4 of the central nervous system. Efferent neurons share many properties of branchial cranial motor neurons, especially the facial branchial motor neurons, both functionally and developmentally, although they do not form a connection with the muscle. In mice, by E10.5, they begin to separate, and by E13.5, they separate completely (Bruce et al., 1997). After that, these efferent nerves grow along existing afferent spiral ganglion neurons to reach their synaptic targets, and, finally, they form synapses to hair cells. These efferent nerve connections to the mammalian sensory epithelium were observed simultaneously as a period of circuit refinement of the afferent neurons. Efferent nerves appear in the inner hair cell region by E16.5 and extend to the outer hair cell region around E18.5 (Bruce et al., 1997). While these stages depend on species, similar patterns have been observed in rats (Bruce et al., 2000), hamsters (Simmons et al., 1996), and gerbils (Rontal & Echteler, 2003). As early as this elongation, choline acetyltransferase (CHAT) expression was observed in the axon terminals of efferent neurons. These efferent nerve connections to hair cells are thought to affect prehearing synaptic activity and influence hearing maturation.

We investigated the efferent nerve development in common marmoset by observing the expression of CHAT, vesicle‐associated membrane protein (VAMP2), and synaptophysin (SYP), which have been used as efferent nerve markers. *CHAT* encodes CHAT, a transferase enzyme responsible for synthesizing acetylcholine, which is a major neurotransmitter of the efferent system. Immunoreactivity for CHAT has been used to identify the inner ear efferent neurons (Eybalin & Pujol, 1987). The *VAMP2* gene encodes VAMP2, also known as synaptobrevin 2. VAMP2 is thought to participate in the presynaptic neurotransmitter release between docking and fusion. The expression of VAMP2 at the terminus of the efferent nerve has been reported (Safieddine & Wenthold, 1999). Previously, the developmental expression pattern of VAMP2 has been reported in the rat embryos (He & Yang, 2011). The *SYP* gene encodes SYP protein, which interacts with the synaptic vesicle protein synaptobrevin. The expression of SYP in efferent neurons has also been reported in both rodents (Gil‐Loyzaga & Pujol, 1988; He & Yang, 2011; Knipper et al., 1995; Safieddine & Wenthold, 1999) and humans (Khalifa et al., 2003).

**FIGURE 17:**
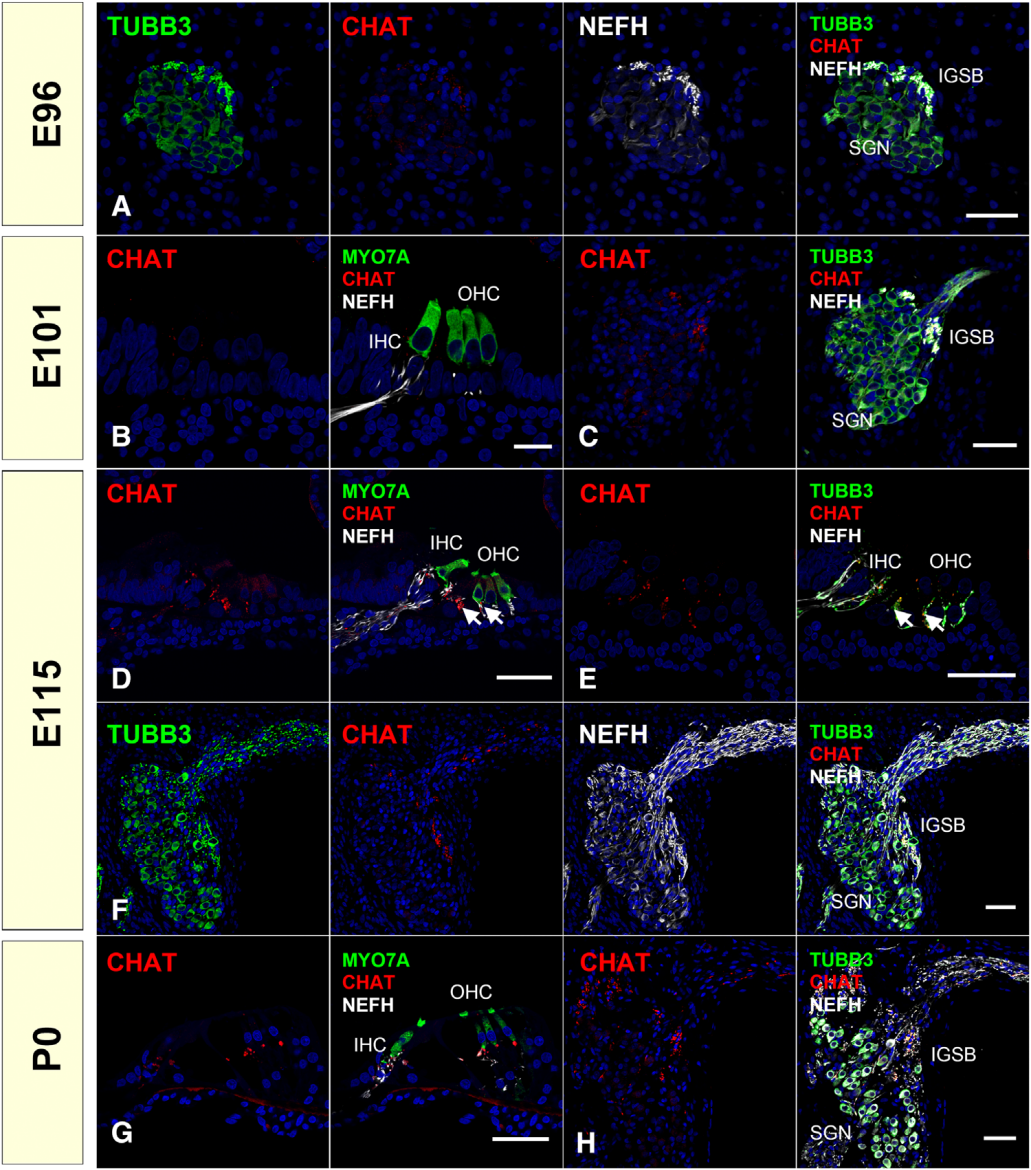
Changes of expression patterns of CHAT during development. (a) At E96, no CHAT expression was observed in the spiral ganglion. (b) and (c) At E101 (b), no expression of CHAT was observed in the organ of Corti. In the spiral ganglion of the common marmoset, CHAT expression comes to be observed in IGSB at E101 (c). (d)–(f) In E115, CHAT expression was detected in the efferent neuronal terminus in the organ of Corti (d). Notably, at this stage, CHAT expression could be observed in neuronal terminus under the inner hair cells and neurite crossing premature Corti tunnels toward first outer hair cells; no CHAT expression could be observed nearby second and third outer hair cells (arrow in d and e). CHAT expression in IGSB is also detected (f). (g) and (h) At P0, CHAT expression is detected in IGSB and the efferent nerve terminus under both inner hair cells and outer hair cells. The nuclei were counterstained with Hoechst (blue). Scale bar: 50 μm in (a), (c)–(h). 50 μm in (b). SGN: spiral ganglion neurons, IGSB: intraganglionic spiral bundle. IHC: inner hair cells, OHC: outer hair cells

**FIGURE 18:**
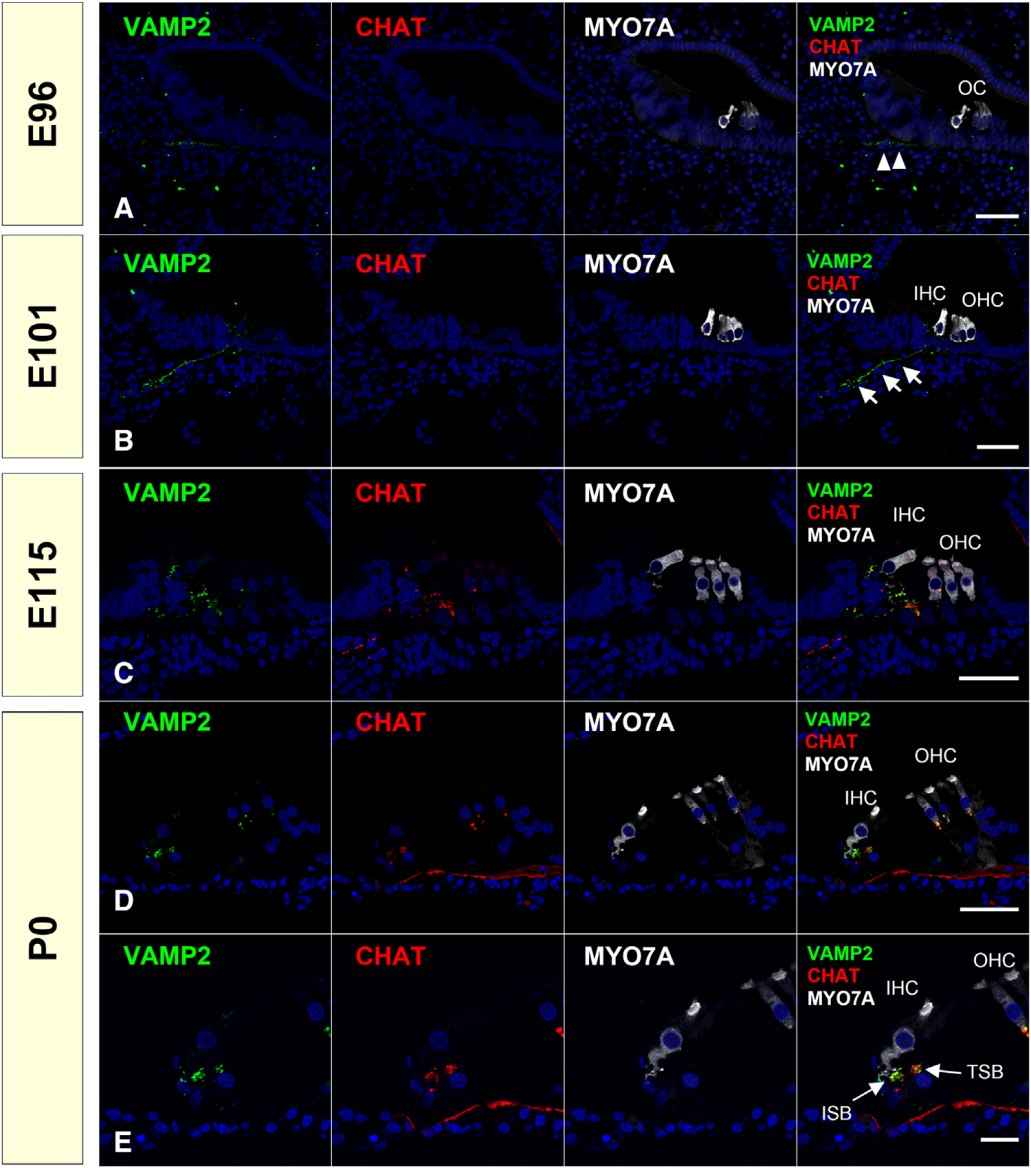
Changes of expression patterns of VAMP2 during development. (a) At E96, slight VAMP2 was observed in the neurons toward the cochlear duct (arrowhead in a). At this stage, no VAMP2 expression was observed in the sensory epithelium. (b) At E101, VAMP2 expression was detected in the efferent neurons (arrow in b), while no CHAT expression was observed. (c) Coexpression of VAMP2 and CHAT was observed in the efferent neurons in the organ of Corti. (d) VAMP2 expression was observed in the CHAT positive efferent neurons at P0. Expression of VAMP2 in the ISB and TSB was also observed. The nuclei were counterstained with Hoechst (blue). Scale bar: 50 μm in (a)–(d). 20 μm in (e). OC: organ of Corti, IHC: inner hair cells, OHC: outer hair cells, ISB: Inner spiral bundle, TSB: Tunnel spiral bundle

**FIGURE 19:**
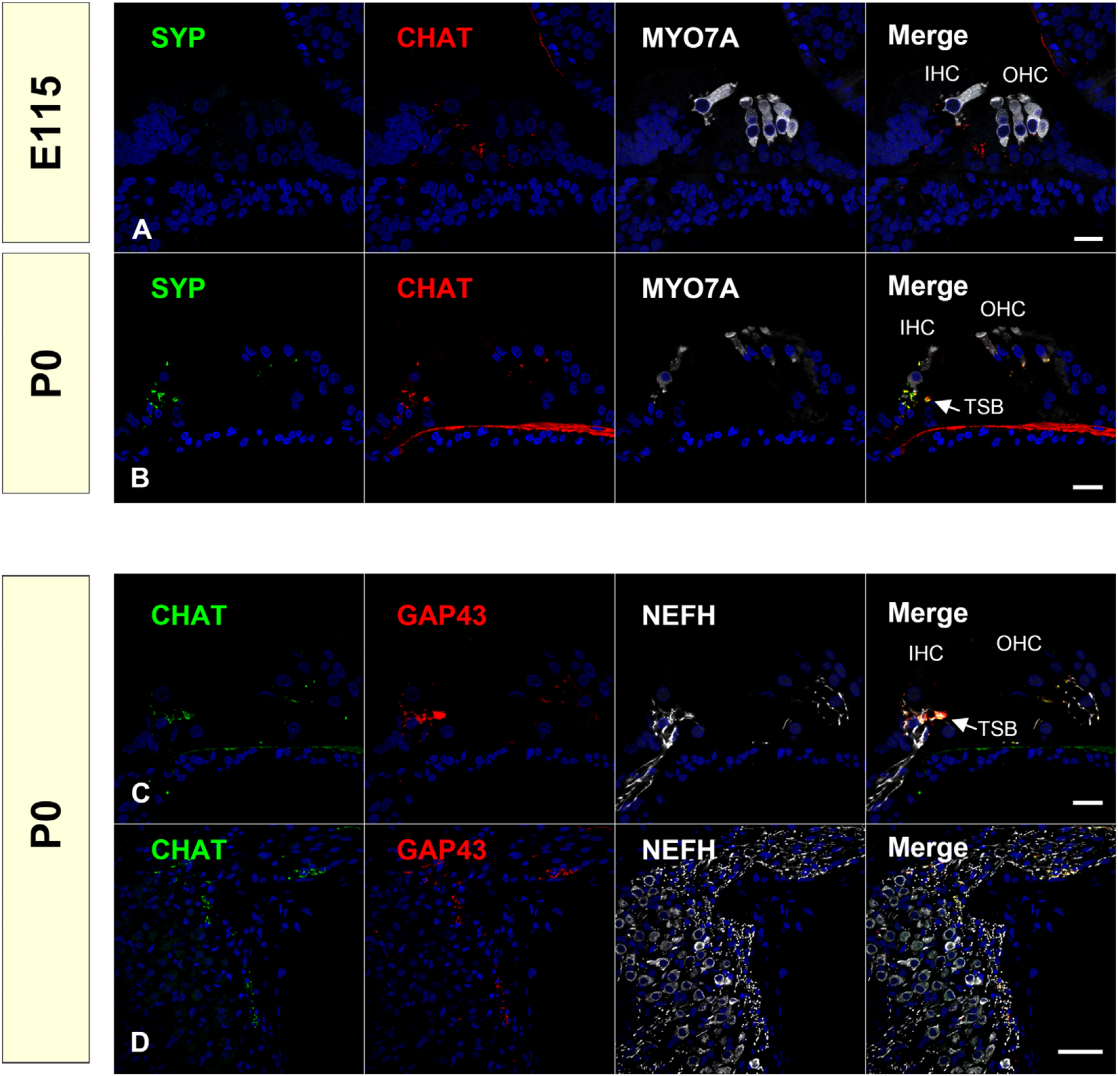
Expression patterns of SYP and GAP43 in the efferent neurons. (a) No SYP expression was observed at E101. (b) SYP expression was observed in the efferent neurons at P0. (c) and (d) GAP43 expression was observed in the efferent neurons at birth. The nuclei were counterstained with Hoechst (blue). Scale bar: 20 μm in (a)–(c). 50 μm in (d). IHC: inner hair cells, OHC: outer hair cells, TSB: Tunnel spiral bundle

In common marmosets, VAMP2 expression precedes the expression of CHAT. While CHAT expression was observed only in IGSB and no expression was observed in efferent neurites at E101 (Figure 17), slight VAMP2 expression was first observed in the innervating efferent neurites at E96, and it was strong at E101 (Figure 18a,b). At E115 and P0, VAMP2 expression was colocalized with CHAT expression observed in the axon terminals of efferent neurons (Figure 18c–e). SYP expression during the latest phase of the developmental stage. SYP expression was observed in the CHAT‐positive termini of the efferent neurons in P0, whereas its expression could not be detected at E115 (Figure 19).

Our observations indicated that efferent nerve innervation to the hair cells occurred around E101 and E115 in the common marmoset, followed by afferent nerve innervation, as seen in other rodent models. This efferent nerve innervation precedes the refinement and pruning of the afferent nerve, which is observed after E115 (Hosoya et al., 2021). Knipper et al. (1995) reported that in efferents projecting to outer hair cells, GAP43 was downregulated at around P14, while in efferents projecting to inner hair cells, GAP43 remained upregulated even beyond P18. In common marmosets, GAP43 expression in CHAT‐positive efferent projections was observed (Figure 19c,d).

Developmental changes in the expression patterns of CHAT in the organ of Corti have been reported in several rodent models (L. C. Huang et al., 2007; Knipper et al., 1995; Merchan Perez et al., 1994; Simmons et al., 1998; Sobkowicz & Emmerling, 1989). These previous reports indicated that in rodents CHAT expression in the efferent nerve is observed around birth, followed by the expression of both VAMP2 and SYP. CHAT expression in the efferent system of the cochlea of humans has been reported (Schrott‐Fischer et al., 2007). In humans, it is not until GW20–22 that efferent contacts are made with the base of outer hair cells, forming axosomatic synapses (Lavigne‐Rebillard & Pujol, 1988; Pujol & Lavigne‐Rebillard, 1985). Detailed VAMP2 and SYT expression patterns in the efferent nerve innervation periods have not been reported in humans.

**FIGURE 20:**
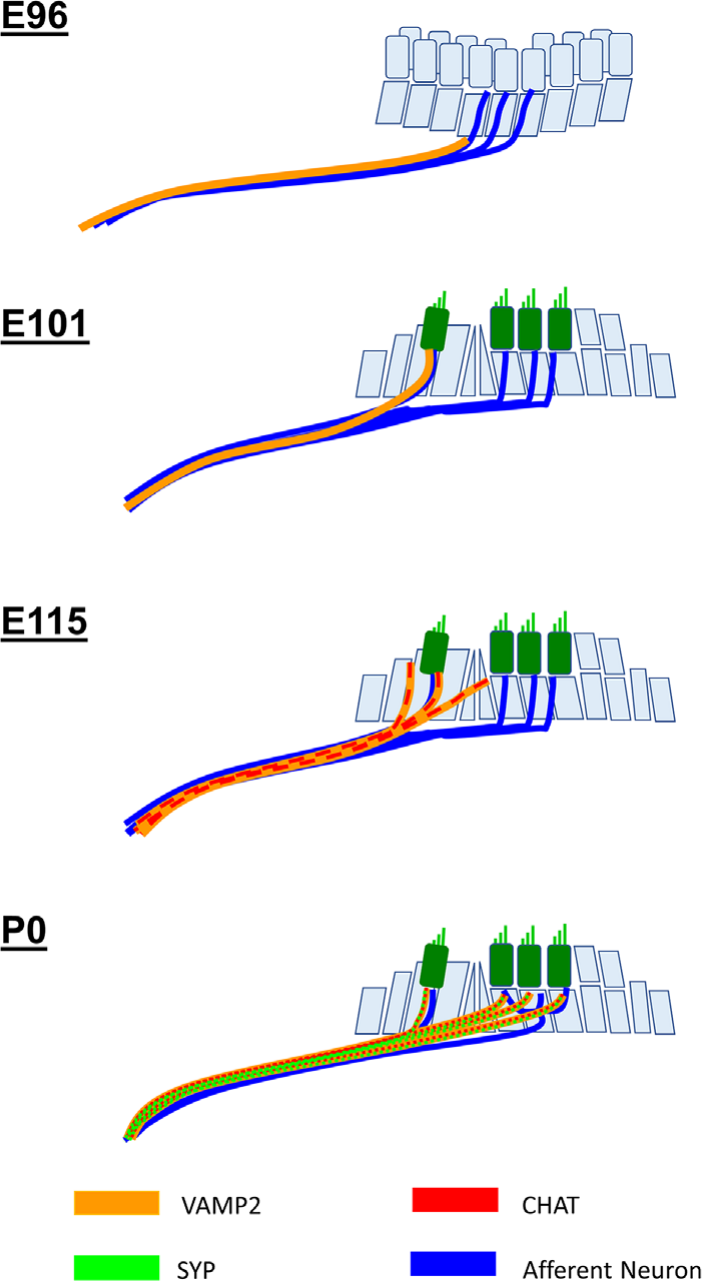
Schematic diagram of the expression pattern of efferent nerve markers in the cochlear development of common marmoset. In the efferent nerve's development of the common marmoset, VAMP2 expression was first observed at E96, followed by CHAT expression at E115 and SYP expression at P0. Crossing of the efferent nerve over the tunnel of Corti was observed around E115

Morphologically, the common marmoset E115 is equivalent to P9 in mice and GW20 in humans (Hosoya et al., 2021). Based on previous reports, the timing of CHAT expression in the organ of Corti was well preserved between the species. Additionally, in the common marmoset, SYP expression followed CHAT expression in rodents, while the timing of VAMP2 expression was distinct in the primate (Figure 20). Whether this particular expression timing of VAMP2 is common marmoset‐specific or primate‐specific has not been clarified, and detailed observation of this period of a human fetus is needed to further understand the implications of these results.

### 2.8 Expression patterns of synaptic vesicle exocytosis related proteins: OTOF, SYTs, and SNAP25

Finally, we examined the expression pattern of genes related to synaptic vesicle exocytosis to estimate the developmental maturation of ribbon synapses in the common marmoset. The exocytosis of synaptic vesicles from the presynaptic membrane to the synaptic space is essential for synaptic transmission. This synaptic vesicle exocytosis involves two essential processes: docking of a synaptic vesicle with the presynaptic plasma membrane and subsequent release of the neurotransmitter caused by fusing the vesicle membrane and presynaptic plasma membrane. SNARE (soluble NSF attachment protein receptor complex) proteins are critical molecules in this docking step. Otoferlin and synaptotagmins can trigger this fusion of the vesicle membrane with the plasma membrane in response to Ca^2+^ influx into the cells following depolarization. Changes in the expression patterns of these synaptic vesicle exocytosis‐related proteins have been reported during hair cell maturation. However, the mechanisms of exocytosis of synaptic vesicles in hair cells differ from those in conventional neurons and are not fully understood.Nouvian et al. (2011) reported that protein products of neuronal SNAREs were not present in the hair cells by observing two postnatal weeks in mice. They suggested the possible existence of other SNARE proteins for synaptic vesicle exocytosis in neurons, which remain to be identified (Nouvian et al., 2011). Mature inner hair cell ribbon synapses also lack several proteins critical for vesicle fusion in synapses of the central nervous system, including SYT1 (Beurg et al., 2010; Safieddine & Wenthold, 1999), while transient expression of SYT1 in the inner hair cells has been reported (S. L. Johnson et al., 2010).

**FIGURE 21:**
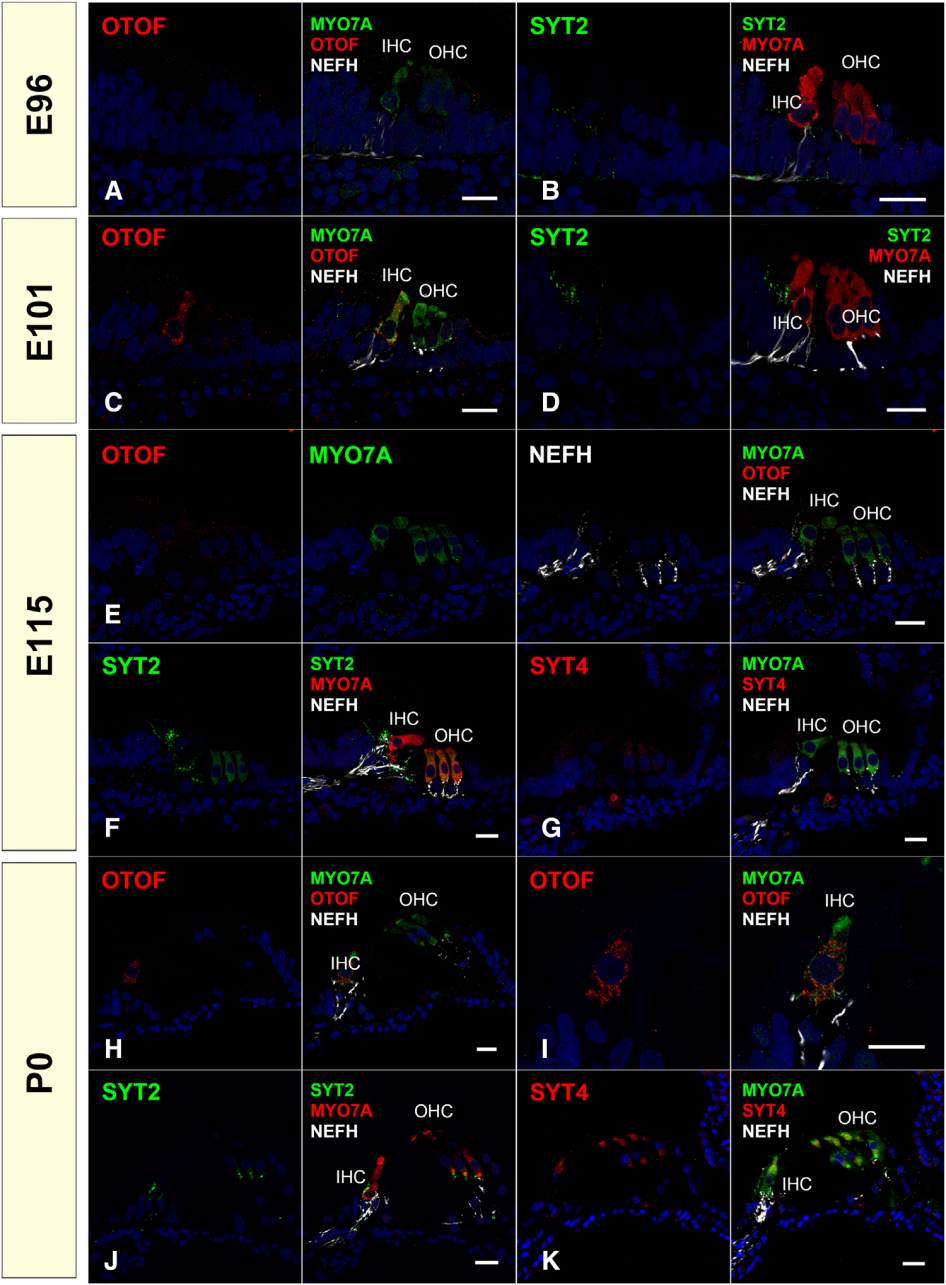
Expression patterns of OTOF, SYT2, and SYT4 during development. (a) At E96, no OTOF expression was observed in the hair cells. (b) At E96, no SYT2 expression was observed. (c) At E101, OTOF expression was observed in the inner hair cells. (d) SYT2 expression was observed in the neuronal terminus around the inner hair cells in the cochlea of E101. No SYT2 expression in the hair cells was detected. (e) At E115, no OTOF expression was detectable. (f) At E115, SYT2 expression in the outer hair cells was detected. At this stage, SYT2 expression was also detected in the neurite terminus near the pillar cells and the terminus under the inner hair cells. (g) No SYT4 expression was detected at E115. (h) and (i) At P0, OTOF expression was observed in the inner hair cells. No expression was observed in the outer hair cells. (j) At P0, no SYT2 expression could be detected in both hair cells, while its expression was observed in the axon terminal under both inner hair cells and outer hair cells. (k) At P0, SYT4 expression in both inner and outer hair cells was detected. The nuclei were counterstained with Hoechst (blue). Scale bar: 20 μm. IHC: inner hair cells, OHC: outer hair cells.

**FIGURE 22:**
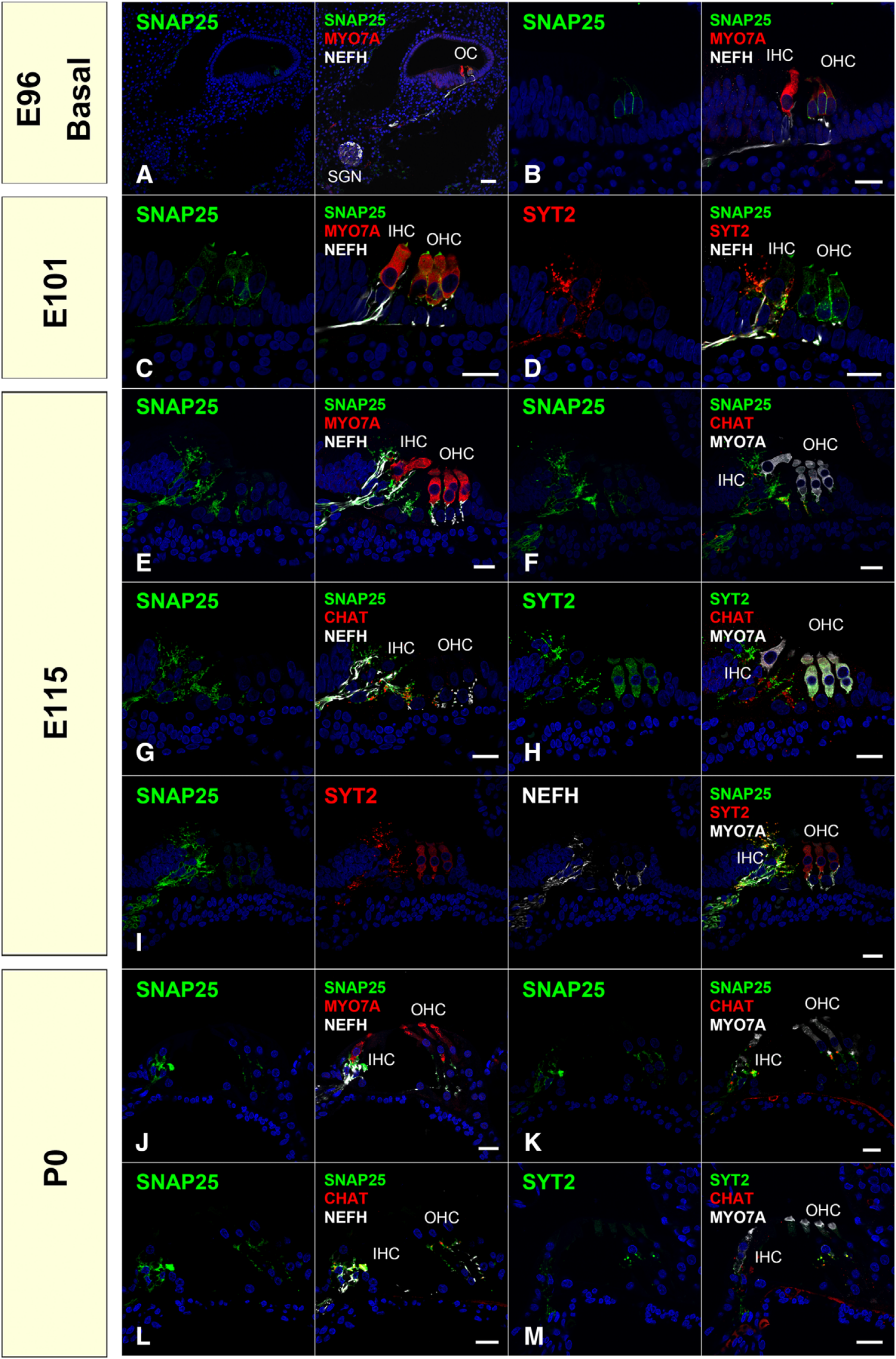
Expression patterns of SNAP25 during development and relationships of the expression pattern of SNAP25, SYT2, and CHAT. (a) and (b) SNAP25 expression was detected in outer hair cells of the basal turn at E96, while no expression was observed in inner hair cells. no expression was observed in spiral ganglion neurons. (c) At E101, SNAP25 expression came to be detected in both inner and outer hair cells. SNAP25 expression was also detected in the neurite around the inner hair cells. (d) At E101, Coexpression of SYT2 and SNAP25 was observed in the organ of Corti. (e) At E115, SNAP25 expression in the hair cells was diminished, and SNAP25 expression in the neurite terminus was more dominantly observed. (f) and (g) At E115, SNAP25 expression was observed in the CHAT‐positive efferent nerve. (h) At E115, SYT2 expression was observed in CHAT‐positive efferent neurons. (i) At E115, coexpression of the SYT2 and SNAP25 was observed in the organ of Corti. (j) At P0, expression of SNAP25 was observed in both synaptic termini of both afferent and efferent neurons. (k) and (l). At P0, in the organ of Corti, SNAP25 expression was observed in CHAT‐ positive TSB and CHAT‐negative OSB: SNAP25 expression was detected in both efferent and afferent systems. (m) At P0, SYT2 expression was observed in CHAT‐positive efferent neurons. The nuclei were counterstained with Hoechst (blue). Scale bar: 50 μm in (a), 20 μm in (b)–(m). SGN: spiral ganglion neuron, OC: organ of Corti, IHC: inner hair cells, OHC: outer hair cells

We examined the expression patterns of otoferlin (OTOF), synaptotagmins (SYT2 and SYT4), and synaptosome‐associated protein 25 (SNAP25) (Figures 21 and 22). OTOF is a protein encoded by the *OTOF* gene. Mutations in this gene cause neurosensory nonsyndromic recessive deafness (DFNB9). OTOF acts as the major Ca^2+^ sensor that triggers synaptic vesicle fusion with the plasma membrane in the inner hair cells (Roux et al., 2006), setting the rates of primed vesicle fusion with the presynaptic plasma membrane and synaptic vesicle pool replenishment in the active zone of inner hair cells (Michalski et al., 2017). In DFNB9 patients, this essential OTOF that mediates Ca^2+^ sensing followed by synaptic vesicle fusion is disturbed, and the patients show auditory neuropathy causing congenital hearing loss.

SYTs, which are a large family of transmembrane proteins containing tandem Ca^2+^‐binding C2‐domains, confer Ca^2+^ sensitivity to SNARE‐dependent vesicle fusion in the central nervous system (Chapman, 2008). Ca^2+^‐bound SYTs binding to the SNARE complex causes vesicle fusion and exocytosis of neurotransmitters. In the cochlea, their functions have been investigated in ribbon synapse formation, especially their relationship with OTOF, which is the main Ca^2+^ sensor in the ribbon synapse of cochlear hair cells. In this synaptotagmin family, the expression of several SYTs has been reported in the cochlea and some of them have been reported to exhibit distinct changes in their expression patterns as maturation proceeds. Transient expression of SYT1 in inner hair cells has been reported (S. L. Johnson et al., 2010). The expression of SYT2 has been reported in adult inner hair cells in mice, although it has not been observed in immature hair cells (S. L. Johnson et al., 2010). The expression of SYT4 in mature hair cells has been reported, and SYT4 is involved in the developmental transition of exocytosis from nonlinear to linear Ca^2+^ dependence (S. L. Johnson et al., 2010).

*SNAP25* encodes SNAP25 protein, a component of the trans‐SNARE complex, which accounts for membrane fusion specificity and directly executes fusion by forming a tight complex that brings the synaptic vesicle and plasma membranes together. SNAP25 is an essential component of SNARE complexes that drive fast Ca^2+^‐dependent exocytosis. SNAP25 directly interacts with OTOF (Ramakrishnan et al., 2009). While SNAP25 is a critical component in most neurons, its expression has been controversial in mature hair cells (Nouvian et al., 2011; Safieddine & Wenthold, 1999), and SNAP25 is not required for apparent exocytosis at the hair cell ribbon synapses (Nouvian et al., 2011). Therefore, it is thought that exocytosis in inner hair cells is unconventional and may operate independently of neuronal SNAREs.

**FIGURE 23:**
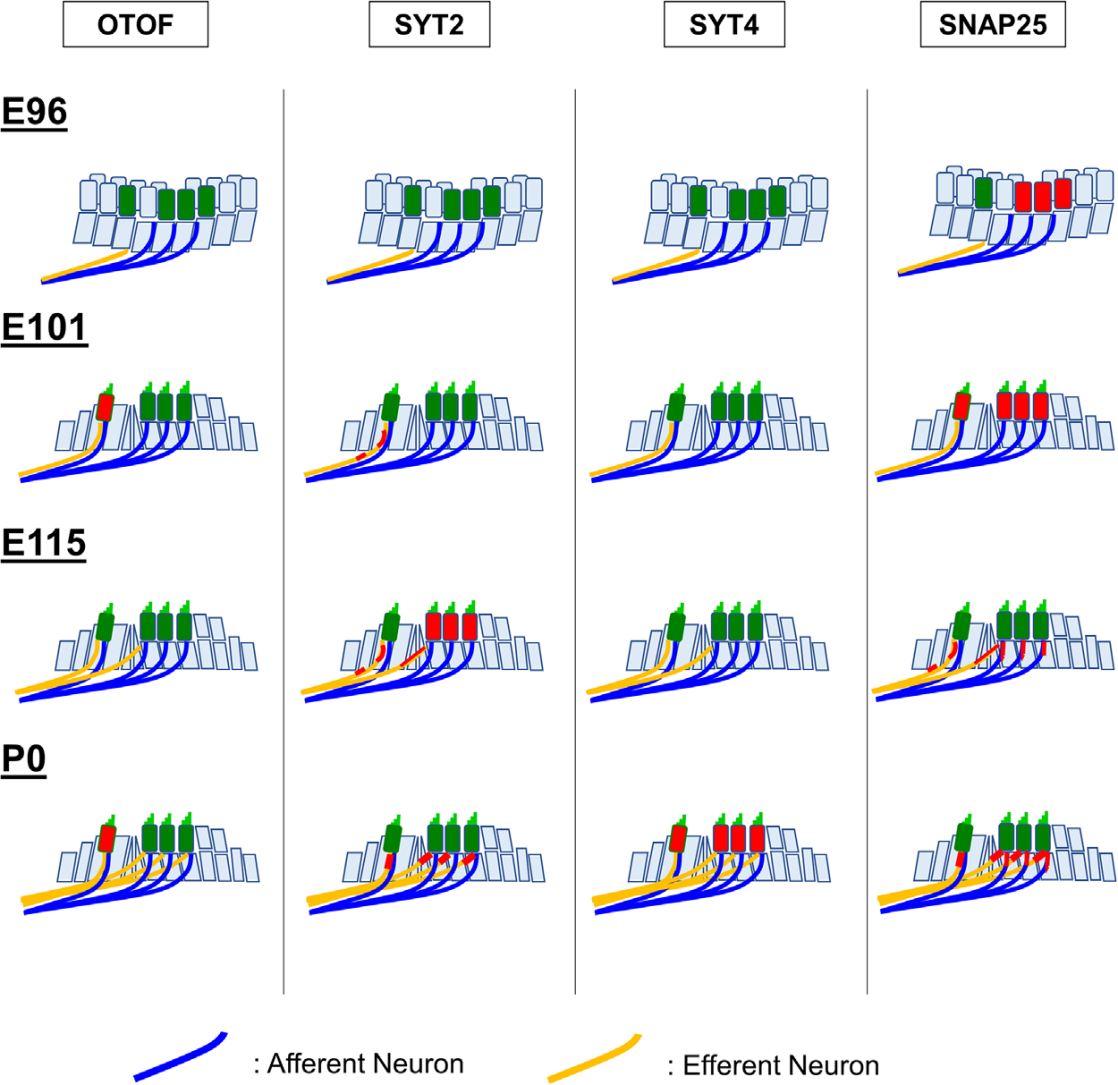
Schematic diagram of the expression pattern of SNARE proteins in the cochlear development of common marmoset

In the common marmoset, OTOF expression was first detected at E101 and decreased during the developmental process (Figure 21). SYT2 in hair cells showed only transient expression in the outer hair cells at E115 (Figure 21). While SYT4 expression was not detected until E115, its expression was observed in both the inner and outer hair cells at P0 (Figure 21). SNAP25 expression was observed in immature outer hair cells at E96, followed by inner hair cells at E101. However, its expression was not detected in either hair cells after E115 (Figure 22). These proteins related to synaptic vesicle exocytosis are also expressed in efferent nerve terminals marked by CHAT (Figures 22 and 23).

**FIGURE 24:**
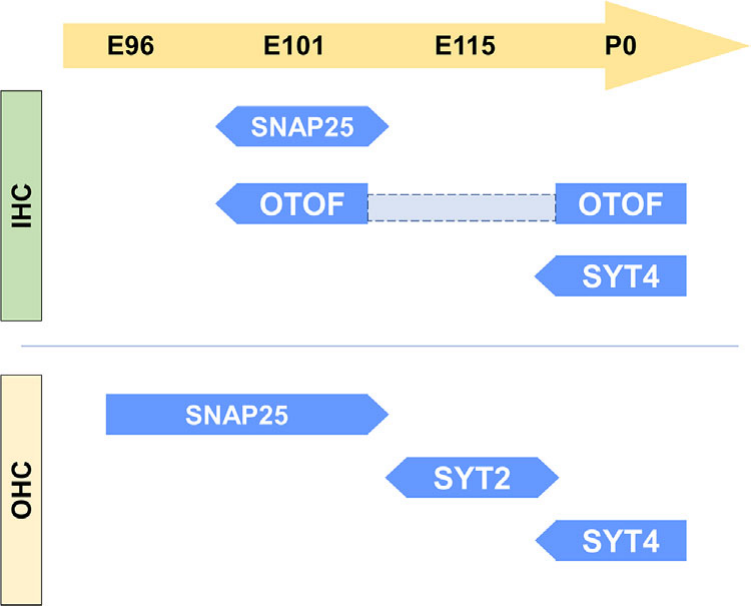
Schematic diagram of the expression pattern of SNARE proteins in the hair cells during development

The present observation in the common marmoset suggests that the fundamental molecules of synaptic vesicle exocytosis are complexly changing as a developmental process in this primate (Figure 24). For example, it is possible that premature hair cells transiently use SNAP25 for vesicle exocytosis before obtaining mature type exocytosis by OTOF. SNAP25 and SYT2 were also transiently expressed in the outer hair cells during the developmental time course of neurite innervation to the outer hair cells, while its expression was diminished entirely at birth. This observation indicates the possible exocytosis of the neurotransmitter from the immature outer hair cells, which can influence synaptic formation between the outer hair cells and spiral ganglion neurons. Our observations indicated that the expression pattern of SYT4 was well preserved between primates and rodents, while SYT2 expression showed species differences.

### 2.9 The common marmoset as an animal model for cochlear neuronal developmental study

**FIGURE 25:**
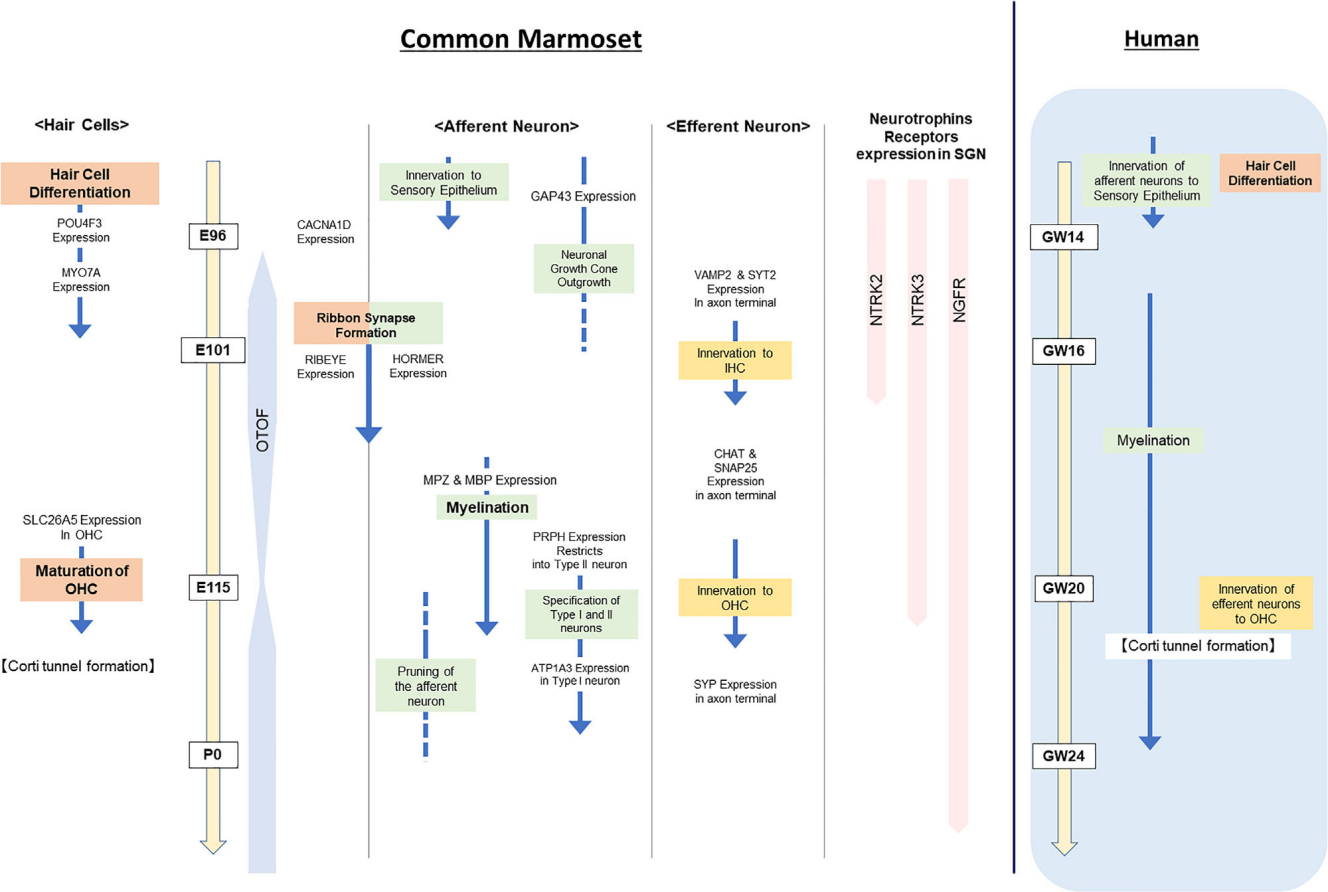
Timeline of cochlear neuronal development of the common marmoset

The time course of cochlear development in the common marmoset is summarized in Figure 25, in which the essential developmental steps of cochlear neuronal development, including ribbon synapse formation, myelination, and efferent nerve innervation, are shown. The developmental steps of the organ of Corti which were reported previously, specifically hair cell differentiation and Corti tunnel formation (Hosoya et al., 2021) and the key time points of human cochlear development, are also shown in the figure. This schema indicates the temporal relationship between crucial steps. For example, the specification of types I and II neurons was observed simultaneously with the maturation of outer hair cells. In another example, the onset of myelination in spiral ganglion neurons preceded the pruning of afferent neurons. This comprehensive neuronal developmental time course of the common marmoset obtained from this study would be useful for further studies using this model animal. In future studies, the appropriate point of the developmental stage can be chosen depending on the purpose of the research. This time course could also be useful for human fetal studies, despite the current minimal opportunities for its use. This schema can guide researchers on how best to utilize the rare opportunities for human fetal studies by allowing researchers to determine what should be observed depending on gestation weeks in advance.

Investigation of the developmental process of the common marmoset is also useful for regenerative studies and age‐related hearing loss studies. To clarify, the detailed time course of this primate can provide us with clues for the regeneration of human hearing. Recapitulation of elements of embryonic development in adults is considered a promising method for organ regeneration. For hearing regeneration, the synapse between the hair cells and spiral ganglion neurons, which can be lost due to aging, could be one of the potential targets for investigation. The central belief in regenerative biology is that the processes that control the development of tissue formation control its regeneration: processes regulating cochlear neuronal development are likely to provide critical insights into neuronal repair in the aging cochlea. This comprehensive analysis of primate neuronal development in the cochlea could provide unprecedented insight into the regeneration of hearing loss.

The common marmoset is poised to be a standard nonhuman primate aging model, and it is also hoped to be an age‐related hearing loss model (Tardif et al., 2011). Primary neuronal degeneration in spiral ganglion neurons is thought to be one of the leading causes of age‐related hearing loss. During this degeneration, changes in the expression patterns of several genes involved in the development process were observed. For example, it was reported that a progressive decrease in SYP was found in hair cells with aging, and the cochlea aging process clearly affects presynaptic membrane proteins of efferent ending fibers (Bartolome et al., 2009). A progressive decrease in neurotrophins with aging has also been reported (Ruttiger et al., 2007). The developmental knowledge obtained in this study could enable us to examine aged marmoset cochlea, comparing the developmental and aging processes in future research.

Moreover, the cochlear developmental time course of the common marmoset is more similar to that of humans than rodents; compared to rodents, its development takes three times longer. It has been reported that this longer developmental time course is suitable for detecting the expression patterns of several genes. For example, we have previously reported that NKCC1, a stria vascularis marker, could be transiently observed in the organ of Corti (Hosoya et al., 2021). In the present study, we have also reported several transient expression patterns that have not been reported in rodent models. Whether these temporary expression patterns are primate specific or can be detected in rodents by an investigation with higher temporal resolutions remains unclear. However, it would remain true that this longer developmental time course is suitable for detecting transient expression patterns of several genes, and it will add novel insights to our knowledge of cochlear development, especially for research on genetic hearing loss without the phenotypic divergence between rodent models and human diseases.

Our observations included important timelines of cochlear neuronal development in this model animal and revealed several differences between primates and rodents from the viewpoint of cochlear development. Our investigation showed the usefulness of these model animals and made possibilities for further developmental studies using this model animal. These findings would be useful for further analysis of neuronal development or functional analysis of the primate inner ear as an alternative to humans in future studies.

## 3 CONCLUSION

We investigated the cochlear development of a nonhuman primate, the common marmoset. We determined the time course of the key developmental stages of neuronal development in this primate model. In addition, we clarified that the similarities and differences of the expression profile of developmental proteins in the cochlea between the previously established rodent model animals and the primate model. Our new developmental data provide a useful tool for studying primate and human cochlear neuronal development.

## 4 MATERIALS AND METHODS

### 4.1 Specimens

Cadaverous fixed head samples of common marmosets at E96 (*n* = 5), E101 (*n* = 4), E115 (*n* = 4), and P0 (*n* = 5) were kindly provided by Ayako Murayama and the Central Institute for Experimental Animals (CIEA).

The animal experiments were approved by the Animal Experiment Committee of Keio University (number: 11006, 08020) and RIKEN (H30‐2‐214(3)) and were conducted following the guidelines of the National Institutes of Health and the Ministry of Education, Culture, Sports, Science and Technology of Japan.

### 4.2 Tissue preparation

The temporal bone region of each common marmoset embryo was dissected and fixed with 4% paraformaldehyde in PBS for 24 h immediately after euthanasia. P0 specimens were decalcified in decalcifying solution B (Wako, Osaka, Japan) for 1 week and then embedded in Tissue‐Tek O.C.T. compound for cross‐sectioning. Seven micrometer sections were used for the immunohistochemical analysis.

### 4.3 Immunohistochemistry

After a brief wash with PBS, the sections were heated (80°C) in 10 μM citrate buffer (pH 6) for 15 min. After a brief wash, the sections were preblocked for 1 h at room temperature in 10% normal serum in PBS with 0.01% Tween 20, incubated with primary antibodies at 4°C overnight, and then incubated with Alexa Fluor‐conjugated secondary antibodies for 60 min at room temperature. Nuclei were counterstained with Hoechst 33258.

For the CACNA1D antibody, sections were heated (60°C) in antigen retrieval buffer (100× Tris‐EDTA buffer, pH 9.0; ab93684, Abcam) for 3–10 min. For the OTOF antibody, sections were heated and the sections were preblocked for 1 h at room temperature in 10% normal serum in PBS without Tween 20.

### 4.4 Antibodies

The primary antibodies used in this study are listed in Table 1.

**TABLE 1:** Antibodies used in this study

Antibody	Host	Isotype	Catalog ID	Provider	Dilution
anti‐POU4F3	mouse	IgG1	sc‐81980	Santa Cruz Biotechnology, Santa Cruz, CA, USA	1:200
anti‐MYOSIN7a	mouse	IgG1	138‐1‐s	DSHB, Iowa City, IA, USA	1:30
anti‐MYOSIN7a	rabbit	IgG	25‐6790	Proteus Biosciences, Ramona, CA, USA	1:100
anti‐GAP43	rabbit	IgG	EP890Y	Abcam, Cambridge, UK	1:1000
anti‐NEFH	chick	IgY	ab4680	Abcam, Cambridge, UK	1:1000
anti‐MAP2	mouse	IgG1	M4403	Merck KGaA, Darmstadt, Germany	1:400
anti‐TUBB	mouse	IgG2b	GTX631836	GeneTex, Irvine, CA, USA	1:1000
anti‐NEFM	mouse	IgG2a	RMO‐270	Thermo Fisher Scientific, Rockford, IL, USA	1:1000
anti‐NEFL	mouse	IgG1	NR‐4	Novus, St. Charles, MO, USA	1:1000
anti‐MPZ	rabbit	IgG	ab31851	Abcam, Cambridge, UK	1:200
anti‐MBP	rat	IgG	M9434	Merck KGaA, Darmstadt, Germany	1:100
anti‐NGFR	rabbit	IgG	ab52987	Abcam, Cambridge, UK	1:1000
anti‐NTRK2	rabbit	IgG	3593‐30T	BioVision, Milpitas, CA, USA	1:500
anti‐NTRK3	rabbit	IgG	C44H5	Cell Signaling Technology, Danvers, MA, USA	1:1000
anti‐RIBEYE	mouse	IgG1	612044	BD Biosciences, San Jose, CA, USA	1:500
anti‐HOMER1	rabbit	IgG	160 003	Synaptic Systems, Goettingen, Germany	1:500
anti‐CACNA1D	rabbit	IgG	HPA020215	Merck KGaA, Darmstadt, Germany	1:300
anti‐ATP1A3	mouse	IgG1	MA3‐915	Invitrogen, Carlsbad, CA, USA	1:500
anti‐PRPH	rabbit	IgG	AB1530	Merck Millipore, Burlington, MA, USA	1:100
anti‐CALB1	rabbit	IgG	ab11426	Abcam, Cambridge, UK	1:1000
anti‐CALB2	rabbit	IgG	GTX103261	GeneTex, Irvine, CA, USA	1:200
anti‐CHAT	goat	IgG	AB144P	Merck KGaA, Darmstadt, Germany	1:200
anti‐VAMP2	mouse	IgG1	104 211	Synaptic Systems, Goettingen, Germany	1:200
anti‐SYP	mouse	IgG1	s5768	Merck KGaA, Darmstadt, GERMANY	1:300
anti‐OTOF	mouse	IgG1	ab53233	Abcam, Cambridge, UK	1:100
anti‐SYT2	mouse	IgG2a	znp1	DSHB, Iowa City, IA, USA	1:100
anti‐SYT4	rabbit	IgG	18977	IBL, Maebashi, JP	1:1000
anti‐SNAP25	mouse	IgG1	#111 011	Synaptic Systems, Goettingen, GERMANY	1:500

The following secondary antibodies were used: Goat anti‐Rabbit IgG, Alexa Fluor Plus 555 (A32732, 1:500, Invitrogen), Goat anti‐Mouse IgG, Alexa Fluor Plus 488 (A32723, 1:500, Invitrogen), Goat anti‐Mouse IgG1, Alexa 488 (A21121, 1:500, Invitrogen), Goat anti‐Mouse IgG2a, Alexa 555 (A21137, 1:500, Invitrogen), Goat anti‐Mouse IgG2b, Alexa 555 (A21147, 1:500, Invitrogen), Goat anti‐Mouse IgG2b, Alexa 647 (A21242, 1:500, Invitrogen), Goat anti‐Chicken IgY, Alexa Fluor Plus 488 (A32931, 1:500, Invitrogen), Goat anti‐Chicken IgY, Alexa Fluor 647 (A21449, 1:500, Invitrogen), Goat anti‐Rat IgG, Alexa Fluor 647 (ab 150159, 1:500, Abcam), Donkey anti‐Rabbit IgG, Alexa Fluor Plus 647 (A32795, 1:500, Invitrogen), Donkey anti‐Mouse IgG, Alexa Fluor Plus 488 (A32766, 1:500, Invitrogen), Donkey anti‐Goat IgG, Alexa Fluor Plus 488 (A32814, 1:500, Invitrogen), Donkey anti‐Goat IgG, Alexa Fluor Plus 555 (A32816, 1:500, Invitrogen), Donkey anti‐Chicken IgY, Alexa Fluor 647 (703‐605‐155, 1:500, Jackson Immuno‐Research).

## CONFLICTS OF INTEREST

H.Okano is a founding scientist and a paid scientific advisory board member of San Bio Co., Ltd. M.H., M.F., and K.O. were founding scientists of Otolink Inc.

## AUTHOR CONTRIBUTIONS

M.H., M.F., A.M., H. Ozawa, H. Okano, and K.O. conceived and designed the experiments. M.H. and M.F. wrote the manuscript. M.H. performed most of the experiments and analyzed the data. All authors read and approved the final version of the manuscript.
